# Supplementary figures and images for: Analyzing the correlation between protein expression and sequence-related features of mRNA and protein in Escherichia coli K-12 MG1655 model
Source: PLoS One. 2024 Feb 7;19(2):e0288526. doi: 10.1371/journal.pone.0288526 (PMC10849221; doi:10.1371/journal.pone.0288526)

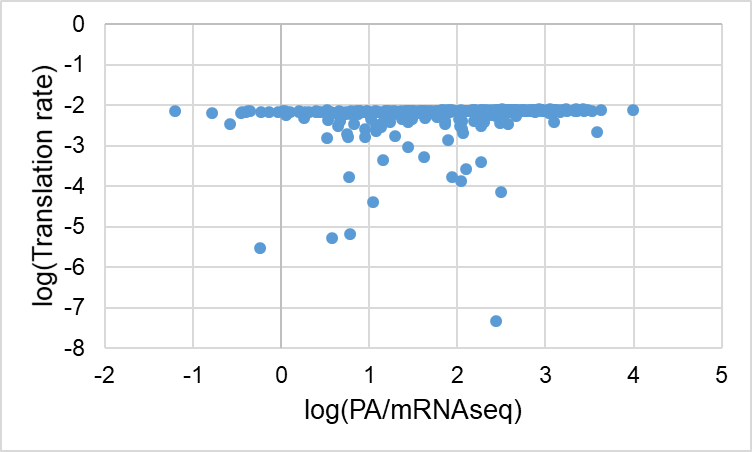

Supplement: S1 Fig — The dataset used here was based on the publication paper of the Transim model (Tuller et al., 2018). (TIF) [file pone.0288526.s001.tif]

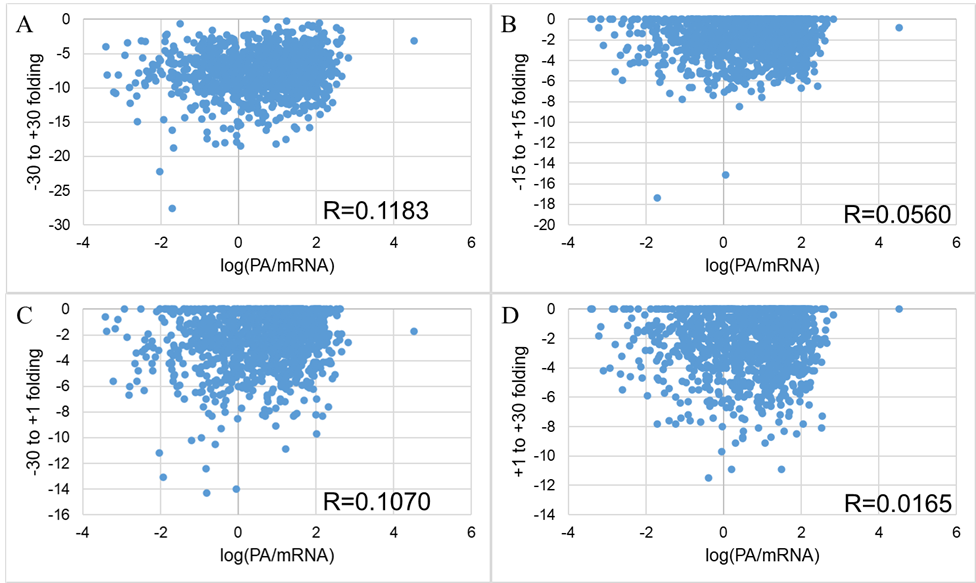

Supplement: S2 Fig — From A to D, the Spearman’s rank coefficient between PA/mRNA and folding energies in the -30 to +30, -15 to +15, -30 to +1, and +30 to +1 regions of the mRNAs. The sequential data here were the same for each analysis, which was 33% less than the total dataset, and hence the difference in Spearman’s coefficient of +1 to +30 folding to PA/mRNA level. (TIF) [file pone.0288526.s002.tif]
