# Supplementary material for: Analyzing the correlation between protein expression and sequence-related features of mRNA and protein in Escherichia coli K-12 MG1655 model
Source: PLoS One. 2024 Feb 7;19(2):e0288526. doi: 10.1371/journal.pone.0288526 (PMC10849221; doi:10.1371/journal.pone.0288526)
Supplement: S1 Table — The first column on the left showed the nucleotide frequency for the first genes in the operons, while the second column on the right showed the frequency from subsequent genes in the operons. The putative Shine-Dalgarno regions were noted in bold, while the color indicated occurrence percentage, with red cells being more used nucleotides and blue cells being less commonly used nucleotides. (DOCX) [file pone.0288526.s003.docx]

|  | First gene in the operon | | | | Subsequent genes in the operon | | | |
| --- | --- | --- | --- | --- | --- | --- | --- | --- |
|  | A | T | G | C | A | T | G | C |
| -15 | 0.37 | 0.26 | 0.19 | 0.18 | 0.33 | 0.35 | 0.16 | 0.15 |
| -14 | 0.36 | 0.23 | 0.21 | 0.19 | 0.34 | 0.21 | 0.21 | 0.24 |
| -13 | 0.38 | 0.23 | 0.24 | 0.16 | 0.40 | 0.18 | 0.27 | 0.15 |
| **-12** | **0.38** | **0.17** | **0.31** | **0.15** | **0.42** | **0.11** | **0.30** | **0.17** |
| **-11** | **0.38** | **0.11** | **0.40** | **0.11** | **0.33** | **0.10** | **0.50** | **0.07** |
| **-10** | **0.31** | **0.12** | **0.50** | **0.06** | **0.31** | **0.07** | **0.57** | **0.05** |
| **-9** | **0.30** | **0.10** | **0.53** | **0.06** | **0.34** | **0.15** | **0.46** | **0.05** |
| **-8** | **0.38** | **0.12** | **0.42** | **0.07** | **0.41** | **0.19** | **0.31** | **0.09** |
| **-7** | **0.38** | **0.18** | **0.34** | **0.10** | **0.36** | **0.25** | **0.28** | **0.11** |
| -6 | 0.36 | 0.25 | 0.24 | 0.14 | 0.37 | 0.24 | 0.21 | 0.17 |
| -5 | 0.34 | 0.29 | 0.19 | 0.18 | 0.36 | 0.27 | 0.18 | 0.20 |
| -4 | 0.35 | 0.28 | 0.18 | 0.20 | 0.44 | 0.19 | 0.18 | 0.20 |
| -3 | 0.41 | 0.19 | 0.22 | 0.19 | 0.35 | 0.25 | 0.20 | 0.20 |
| -2 | 0.26 | 0.32 | 0.13 | 0.29 | 0.25 | 0.33 | 0.14 | 0.28 |
| -1 | 0.23 | 0.35 | 0.14 | 0.28 | 0.56 | 0.23 | 0.09 | 0.11 |
| 1 | 0.89 | 0.02 | 0.08 | 0.00 | 0.90 | 0.02 | 0.08 | 0.00 |
| 2 | 0.00 | 1.00 | 0.00 | 0.00 | 0.00 | 1.00 | 0.00 | 0.00 |
| 3 | 0.00 | 0.00 | 1.00 | 0.00 | 0.00 | 0.00 | 1.00 | 0.00 |
| 4 | 0.47 | 0.16 | 0.21 | 0.16 | 0.47 | 0.20 | 0.20 | 0.14 |
| 5 | 0.36 | 0.18 | 0.16 | 0.30 | 0.36 | 0.17 | 0.14 | 0.33 |
| 6 | 0.35 | 0.31 | 0.15 | 0.18 | 0.34 | 0.31 | 0.17 | 0.18 |
| 7 | 0.46 | 0.12 | 0.22 | 0.20 | 0.41 | 0.16 | 0.22 | 0.21 |
| 8 | 0.45 | 0.23 | 0.13 | 0.20 | 0.43 | 0.26 | 0.14 | 0.18 |
| 9 | 0.31 | 0.28 | 0.21 | 0.19 | 0.35 | 0.31 | 0.19 | 0.15 |
| 10 | 0.39 | 0.18 | 0.20 | 0.22 | 0.32 | 0.23 | 0.21 | 0.25 |
| 11 | 0.33 | 0.32 | 0.12 | 0.23 | 0.33 | 0.30 | 0.12 | 0.25 |
| 12 | 0.34 | 0.30 | 0.16 | 0.20 | 0.35 | 0.33 | 0.15 | 0.17 |
| 13 | 0.36 | 0.18 | 0.24 | 0.21 | 0.37 | 0.16 | 0.24 | 0.22 |
| 14 | 0.34 | 0.33 | 0.11 | 0.22 | 0.27 | 0.43 | 0.10 | 0.21 |
| 15 | 0.27 | 0.31 | 0.19 | 0.23 | 0.21 | 0.38 | 0.15 | 0.25 |
