# Supplementary material for: Analyzing the correlation between protein expression and sequence-related features of mRNA and protein in Escherichia coli K-12 MG1655 model
Source: PLoS One. 2024 Feb 7;19(2):e0288526. doi: 10.1371/journal.pone.0288526 (PMC10849221; doi:10.1371/journal.pone.0288526)
Supplement: S2 Table — (DOCX) [file pone.0288526.s004.docx]

| **PA/mRNA** |  |  |  |  |  |  |  |  |  | **Spearman** |
| --- | --- | --- | --- | --- | --- | --- | --- | --- | --- | --- |
| Base |  |  |  |  |  |  |  |  |  | 0.617832 |
| +1 to +30 |  |  |  |  |  |  |  |  |  | 0.617996 |
| CAI |  |  |  |  |  |  |  |  |  | 0.606927 |
| Half-life |  |  |  |  |  |  |  |  |  | 0.61572 |
| Instability |  |  |  |  |  |  |  |  |  | 0.614062 |
| Start |  |  |  |  |  |  |  |  |  | 0.618951 |
| Stop |  |  |  |  |  |  |  |  |  | 0.618635 |
| Type |  |  |  |  |  |  |  |  |  | 0.622554 |
| length |  |  |  |  |  |  |  |  |  | 0.6186 |
| tAI |  |  |  |  |  |  |  |  |  | 0.61733 |
| uAUG |  |  |  |  |  |  |  |  |  | 0.614932 |
| +1 to +30 | CAI |  |  |  |  |  |  |  |  | 0.605958 |
| +1 to +30 | Half-life |  |  |  |  |  |  |  |  | 0.615654 |
| +1 to +30 | Instability |  |  |  |  |  |  |  |  | 0.614694 |
| +1 to +30 | Start |  |  |  |  |  |  |  |  | 0.619119 |
| +1 to +30 | Stop |  |  |  |  |  |  |  |  | 0.618538 |
| +1 to +30 | Type |  |  |  |  |  |  |  |  | 0.622127 |
| +1 to +30 | length |  |  |  |  |  |  |  |  | 0.61903 |
| +1 to +30 | tAI |  |  |  |  |  |  |  |  | 0.617266 |
| +1 to +30 | uAUG |  |  |  |  |  |  |  |  | 0.615185 |
| CAI | Half-life |  |  |  |  |  |  |  |  | 0.603699 |
| CAI | Instability |  |  |  |  |  |  |  |  | 0.604938 |
| CAI | Start |  |  |  |  |  |  |  |  | 0.6086 |
| CAI | Stop |  |  |  |  |  |  |  |  | 0.610423 |
| CAI | Type |  |  |  |  |  |  |  |  | 0.613987 |
| CAI | length |  |  |  |  |  |  |  |  | 0.611053 |
| CAI | tAI |  |  |  |  |  |  |  |  | 0.607768 |
| CAI | uAUG |  |  |  |  |  |  |  |  | 0.603879 |
| Half-life | Instability |  |  |  |  |  |  |  |  | 0.611519 |
| Half-life | Start |  |  |  |  |  |  |  |  | 0.616547 |
| Half-life | Stop |  |  |  |  |  |  |  |  | 0.616235 |
| Half-life | Type |  |  |  |  |  |  |  |  | 0.620869 |
| Half-life | length |  |  |  |  |  |  |  |  | 0.618157 |
| Half-life | tAI |  |  |  |  |  |  |  |  | 0.615076 |
| Half-life | uAUG |  |  |  |  |  |  |  |  | 0.613426 |
| Instability | Start |  |  |  |  |  |  |  |  | 0.615441 |
| Instability | Stop |  |  |  |  |  |  |  |  | 0.615559 |
| Instability | Type |  |  |  |  |  |  |  |  | 0.619264 |
| Instability | length |  |  |  |  |  |  |  |  | 0.61321 |
| Instability | tAI |  |  |  |  |  |  |  |  | 0.612617 |
| Instability | uAUG |  |  |  |  |  |  |  |  | 0.612036 |
| Start | Stop |  |  |  |  |  |  |  |  | 0.619776 |
| Start | Type |  |  |  |  |  |  |  |  | 0.622881 |
| Start | length |  |  |  |  |  |  |  |  | 0.620749 |
| Start | tAI |  |  |  |  |  |  |  |  | 0.619364 |
| Start | uAUG |  |  |  |  |  |  |  |  | 0.616894 |
| Stop | Type |  |  |  |  |  |  |  |  | 0.622768 |
| Stop | length |  |  |  |  |  |  |  |  | 0.620626 |
| Stop | tAI |  |  |  |  |  |  |  |  | 0.617917 |
| Stop | uAUG |  |  |  |  |  |  |  |  | 0.614875 |
| Type | length |  |  |  |  |  |  |  |  | 0.621768 |
| Type | tAI |  |  |  |  |  |  |  |  | 0.621301 |
| Type | uAUG |  |  |  |  |  |  |  |  | 0.620674 |
| length | tAI |  |  |  |  |  |  |  |  | 0.617379 |
| length | uAUG |  |  |  |  |  |  |  |  | 0.618573 |
| tAI | uAUG |  |  |  |  |  |  |  |  | 0.615418 |
| +1 to +30 | CAI | Half-life |  |  |  |  |  |  |  | 0.602898 |
| +1 to +30 | CAI | Instability |  |  |  |  |  |  |  | 0.604323 |
| +1 to +30 | CAI | Start |  |  |  |  |  |  |  | 0.607778 |
| +1 to +30 | CAI | Stop |  |  |  |  |  |  |  | 0.609719 |
| +1 to +30 | CAI | Type |  |  |  |  |  |  |  | 0.612767 |
| +1 to +30 | CAI | length |  |  |  |  |  |  |  | 0.610941 |
| +1 to +30 | CAI | tAI |  |  |  |  |  |  |  | 0.607479 |
| +1 to +30 | CAI | uAUG |  |  |  |  |  |  |  | 0.603725 |
| +1 to +30 | Half-life | Instability |  |  |  |  |  |  |  | 0.611923 |
| +1 to +30 | Half-life | Start |  |  |  |  |  |  |  | 0.617617 |
| +1 to +30 | Half-life | Stop |  |  |  |  |  |  |  | 0.615845 |
| +1 to +30 | Half-life | Type |  |  |  |  |  |  |  | 0.620565 |
| +1 to +30 | Half-life | length |  |  |  |  |  |  |  | 0.618369 |
| +1 to +30 | Half-life | tAI |  |  |  |  |  |  |  | 0.61561 |
| +1 to +30 | Half-life | uAUG |  |  |  |  |  |  |  | 0.613478 |
| +1 to +30 | Instability | Start |  |  |  |  |  |  |  | 0.616074 |
| +1 to +30 | Instability | Stop |  |  |  |  |  |  |  | 0.615575 |
| +1 to +30 | Instability | Type |  |  |  |  |  |  |  | 0.618899 |
| +1 to +30 | Instability | length |  |  |  |  |  |  |  | 0.613586 |
| +1 to +30 | Instability | tAI |  |  |  |  |  |  |  | 0.613664 |
| +1 to +30 | Instability | uAUG |  |  |  |  |  |  |  | 0.61233 |
| +1 to +30 | Start | Stop |  |  |  |  |  |  |  | 0.619862 |
| +1 to +30 | Start | Type |  |  |  |  |  |  |  | 0.623122 |
| +1 to +30 | Start | length |  |  |  |  |  |  |  | 0.621012 |
| +1 to +30 | Start | tAI |  |  |  |  |  |  |  | 0.619585 |
| +1 to +30 | Start | uAUG |  |  |  |  |  |  |  | 0.616668 |
| +1 to +30 | Stop | Type |  |  |  |  |  |  |  | 0.622092 |
| +1 to +30 | Stop | length |  |  |  |  |  |  |  | 0.620902 |
| +1 to +30 | Stop | tAI |  |  |  |  |  |  |  | 0.617972 |
| +1 to +30 | Stop | uAUG |  |  |  |  |  |  |  | 0.615459 |
| +1 to +30 | Type | length |  |  |  |  |  |  |  | 0.621965 |
| +1 to +30 | Type | tAI |  |  |  |  |  |  |  | 0.621044 |
| +1 to +30 | Type | uAUG |  |  |  |  |  |  |  | 0.620578 |
| +1 to +30 | length | tAI |  |  |  |  |  |  |  | 0.617052 |
| +1 to +30 | length | uAUG |  |  |  |  |  |  |  | 0.618264 |
| +1 to +30 | tAI | uAUG |  |  |  |  |  |  |  | 0.614987 |
| CAI | Half-life | Instability |  |  |  |  |  |  |  | 0.601254 |
| CAI | Half-life | Start |  |  |  |  |  |  |  | 0.603612 |
| CAI | Half-life | Stop |  |  |  |  |  |  |  | 0.603671 |
| CAI | Half-life | Type |  |  |  |  |  |  |  | 0.608318 |
| CAI | Half-life | length |  |  |  |  |  |  |  | 0.610545 |
| CAI | Half-life | tAI |  |  |  |  |  |  |  | 0.599929 |
| CAI | Half-life | uAUG |  |  |  |  |  |  |  | 0.600156 |
| CAI | Instability | Start |  |  |  |  |  |  |  | 0.606536 |
| CAI | Instability | Stop |  |  |  |  |  |  |  | 0.605529 |
| CAI | Instability | Type |  |  |  |  |  |  |  | 0.60766 |
| CAI | Instability | length |  |  |  |  |  |  |  | 0.605639 |
| CAI | Instability | tAI |  |  |  |  |  |  |  | 0.602453 |
| CAI | Instability | uAUG |  |  |  |  |  |  |  | 0.602182 |
| CAI | Start | Stop |  |  |  |  |  |  |  | 0.609172 |
| CAI | Start | Type |  |  |  |  |  |  |  | 0.615291 |
| CAI | Start | length |  |  |  |  |  |  |  | 0.614943 |
| CAI | Start | tAI |  |  |  |  |  |  |  | 0.608859 |
| CAI | Start | uAUG |  |  |  |  |  |  |  | 0.60643 |
| CAI | Stop | Type |  |  |  |  |  |  |  | 0.615588 |
| CAI | Stop | length |  |  |  |  |  |  |  | 0.614113 |
| CAI | Stop | tAI |  |  |  |  |  |  |  | 0.609286 |
| CAI | Stop | uAUG |  |  |  |  |  |  |  | 0.606916 |
| CAI | Type | length |  |  |  |  |  |  |  | 0.617634 |
| CAI | Type | tAI |  |  |  |  |  |  |  | 0.611793 |
| CAI | Type | uAUG |  |  |  |  |  |  |  | 0.610088 |
| CAI | length | tAI |  |  |  |  |  |  |  | 0.606872 |
| CAI | length | uAUG |  |  |  |  |  |  |  | 0.609819 |
| CAI | tAI | uAUG |  |  |  |  |  |  |  | 0.600851 |
| Half-life | Instability | Start |  |  |  |  |  |  |  | 0.613367 |
| Half-life | Instability | Stop |  |  |  |  |  |  |  | 0.613051 |
| Half-life | Instability | Type |  |  |  |  |  |  |  | 0.615399 |
| Half-life | Instability | length |  |  |  |  |  |  |  | 0.6123 |
| Half-life | Instability | tAI |  |  |  |  |  |  |  | 0.610883 |
| Half-life | Instability | uAUG |  |  |  |  |  |  |  | 0.609347 |
| Half-life | Start | Stop |  |  |  |  |  |  |  | 0.617562 |
| Half-life | Start | Type |  |  |  |  |  |  |  | 0.622292 |
| Half-life | Start | length |  |  |  |  |  |  |  | 0.620026 |
| Half-life | Start | tAI |  |  |  |  |  |  |  | 0.617473 |
| Half-life | Start | uAUG |  |  |  |  |  |  |  | 0.61482 |
| Half-life | Stop | Type |  |  |  |  |  |  |  | 0.621638 |
| Half-life | Stop | length |  |  |  |  |  |  |  | 0.619406 |
| Half-life | Stop | tAI |  |  |  |  |  |  |  | 0.615579 |
| Half-life | Stop | uAUG |  |  |  |  |  |  |  | 0.612985 |
| Half-life | Type | length |  |  |  |  |  |  |  | 0.62008 |
| Half-life | Type | tAI |  |  |  |  |  |  |  | 0.620005 |
| Half-life | Type | uAUG |  |  |  |  |  |  |  | 0.616689 |
| Half-life | length | tAI |  |  |  |  |  |  |  | 0.614004 |
| Half-life | length | uAUG |  |  |  |  |  |  |  | 0.616962 |
| Half-life | tAI | uAUG |  |  |  |  |  |  |  | 0.61295 |
| Instability | Start | Stop |  |  |  |  |  |  |  | 0.616941 |
| Instability | Start | Type |  |  |  |  |  |  |  | 0.619161 |
| Instability | Start | length |  |  |  |  |  |  |  | 0.617638 |
| Instability | Start | tAI |  |  |  |  |  |  |  | 0.615504 |
| Instability | Start | uAUG |  |  |  |  |  |  |  | 0.613915 |
| Instability | Stop | Type |  |  |  |  |  |  |  | 0.62016 |
| Instability | Stop | length |  |  |  |  |  |  |  | 0.618928 |
| Instability | Stop | tAI |  |  |  |  |  |  |  | 0.613639 |
| Instability | Stop | uAUG |  |  |  |  |  |  |  | 0.614151 |
| Instability | Type | length |  |  |  |  |  |  |  | 0.61716 |
| Instability | Type | tAI |  |  |  |  |  |  |  | 0.617294 |
| Instability | Type | uAUG |  |  |  |  |  |  |  | 0.616502 |
| Instability | length | tAI |  |  |  |  |  |  |  | 0.61232 |
| Instability | length | uAUG |  |  |  |  |  |  |  | 0.611984 |
| Instability | tAI | uAUG |  |  |  |  |  |  |  | 0.610958 |
| Start | Stop | Type |  |  |  |  |  |  |  | 0.623822 |
| Start | Stop | length |  |  |  |  |  |  |  | 0.622111 |
| Start | Stop | tAI |  |  |  |  |  |  |  | 0.618454 |
| Start | Stop | uAUG |  |  |  |  |  |  |  | 0.617286 |
| Start | Type | length |  |  |  |  |  |  |  | 0.622768 |
| Start | Type | tAI |  |  |  |  |  |  |  | 0.622343 |
| Start | Type | uAUG |  |  |  |  |  |  |  | 0.621594 |
| Start | length | tAI |  |  |  |  |  |  |  | 0.617848 |
| Start | length | uAUG |  |  |  |  |  |  |  | 0.620457 |
| Start | tAI | uAUG |  |  |  |  |  |  |  | 0.61756 |
| Stop | Type | length |  |  |  |  |  |  |  | 0.624524 |
| Stop | Type | tAI |  |  |  |  |  |  |  | 0.622233 |
| Stop | Type | uAUG |  |  |  |  |  |  |  | 0.619872 |
| Stop | length | tAI |  |  |  |  |  |  |  | 0.618742 |
| Stop | length | uAUG |  |  |  |  |  |  |  | 0.619896 |
| Stop | tAI | uAUG |  |  |  |  |  |  |  | 0.616141 |
| Type | length | tAI |  |  |  |  |  |  |  | 0.622073 |
| Type | length | uAUG |  |  |  |  |  |  |  | 0.621596 |
| Type | tAI | uAUG |  |  |  |  |  |  |  | 0.620229 |
| length | tAI | uAUG |  |  |  |  |  |  |  | 0.615397 |
| +1 to +30 | CAI | Half-life | Instability |  |  |  |  |  |  | 0.602035 |
| +1 to +30 | CAI | Half-life | Start |  |  |  |  |  |  | 0.603798 |
| +1 to +30 | CAI | Half-life | Stop |  |  |  |  |  |  | 0.604018 |
| +1 to +30 | CAI | Half-life | Type |  |  |  |  |  |  | 0.607933 |
| +1 to +30 | CAI | Half-life | length |  |  |  |  |  |  | 0.6112 |
| +1 to +30 | CAI | Half-life | tAI |  |  |  |  |  |  | 0.60013 |
| +1 to +30 | CAI | Half-life | uAUG |  |  |  |  |  |  | 0.600262 |
| +1 to +30 | CAI | Instability | Start |  |  |  |  |  |  | 0.605608 |
| +1 to +30 | CAI | Instability | Stop |  |  |  |  |  |  | 0.605328 |
| +1 to +30 | CAI | Instability | Type |  |  |  |  |  |  | 0.607435 |
| +1 to +30 | CAI | Instability | length |  |  |  |  |  |  | 0.606182 |
| +1 to +30 | CAI | Instability | tAI |  |  |  |  |  |  | 0.602193 |
| +1 to +30 | CAI | Instability | uAUG |  |  |  |  |  |  | 0.602133 |
| +1 to +30 | CAI | Start | Stop |  |  |  |  |  |  | 0.609748 |
| +1 to +30 | CAI | Start | Type |  |  |  |  |  |  | 0.614699 |
| +1 to +30 | CAI | Start | length |  |  |  |  |  |  | 0.615405 |
| +1 to +30 | CAI | Start | tAI |  |  |  |  |  |  | 0.609721 |
| +1 to +30 | CAI | Start | uAUG |  |  |  |  |  |  | 0.606372 |
| +1 to +30 | CAI | Stop | Type |  |  |  |  |  |  | 0.615866 |
| +1 to +30 | CAI | Stop | length |  |  |  |  |  |  | 0.61495 |
| +1 to +30 | CAI | Stop | tAI |  |  |  |  |  |  | 0.608574 |
| +1 to +30 | CAI | Stop | uAUG |  |  |  |  |  |  | 0.606963 |
| +1 to +30 | CAI | Type | length |  |  |  |  |  |  | 0.617855 |
| +1 to +30 | CAI | Type | tAI |  |  |  |  |  |  | 0.611964 |
| +1 to +30 | CAI | Type | uAUG |  |  |  |  |  |  | 0.61008 |
| +1 to +30 | CAI | length | tAI |  |  |  |  |  |  | 0.607734 |
| +1 to +30 | CAI | length | uAUG |  |  |  |  |  |  | 0.609247 |
| +1 to +30 | CAI | tAI | uAUG |  |  |  |  |  |  | 0.6017 |
| +1 to +30 | Half-life | Instability | Start |  |  |  |  |  |  | 0.612295 |
| +1 to +30 | Half-life | Instability | Stop |  |  |  |  |  |  | 0.61266 |
| +1 to +30 | Half-life | Instability | Type |  |  |  |  |  |  | 0.616385 |
| +1 to +30 | Half-life | Instability | length |  |  |  |  |  |  | 0.612763 |
| +1 to +30 | Half-life | Instability | tAI |  |  |  |  |  |  | 0.612025 |
| +1 to +30 | Half-life | Instability | uAUG |  |  |  |  |  |  | 0.609816 |
| +1 to +30 | Half-life | Start | Stop |  |  |  |  |  |  | 0.616261 |
| +1 to +30 | Half-life | Start | Type |  |  |  |  |  |  | 0.622524 |
| +1 to +30 | Half-life | Start | length |  |  |  |  |  |  | 0.619824 |
| +1 to +30 | Half-life | Start | tAI |  |  |  |  |  |  | 0.616918 |
| +1 to +30 | Half-life | Start | uAUG |  |  |  |  |  |  | 0.615101 |
| +1 to +30 | Half-life | Stop | Type |  |  |  |  |  |  | 0.620164 |
| +1 to +30 | Half-life | Stop | length |  |  |  |  |  |  | 0.619313 |
| +1 to +30 | Half-life | Stop | tAI |  |  |  |  |  |  | 0.61584 |
| +1 to +30 | Half-life | Stop | uAUG |  |  |  |  |  |  | 0.612539 |
| +1 to +30 | Half-life | Type | length |  |  |  |  |  |  | 0.620246 |
| +1 to +30 | Half-life | Type | tAI |  |  |  |  |  |  | 0.619749 |
| +1 to +30 | Half-life | Type | uAUG |  |  |  |  |  |  | 0.616514 |
| +1 to +30 | Half-life | length | tAI |  |  |  |  |  |  | 0.614041 |
| +1 to +30 | Half-life | length | uAUG |  |  |  |  |  |  | 0.617152 |
| +1 to +30 | Half-life | tAI | uAUG |  |  |  |  |  |  | 0.61207 |
| +1 to +30 | Instability | Start | Stop |  |  |  |  |  |  | 0.616826 |
| +1 to +30 | Instability | Start | Type |  |  |  |  |  |  | 0.618737 |
| +1 to +30 | Instability | Start | length |  |  |  |  |  |  | 0.6185 |
| +1 to +30 | Instability | Start | tAI |  |  |  |  |  |  | 0.615824 |
| +1 to +30 | Instability | Start | uAUG |  |  |  |  |  |  | 0.614174 |
| +1 to +30 | Instability | Stop | Type |  |  |  |  |  |  | 0.619662 |
| +1 to +30 | Instability | Stop | length |  |  |  |  |  |  | 0.618762 |
| +1 to +30 | Instability | Stop | tAI |  |  |  |  |  |  | 0.613581 |
| +1 to +30 | Instability | Stop | uAUG |  |  |  |  |  |  | 0.614442 |
| +1 to +30 | Instability | Type | length |  |  |  |  |  |  | 0.617229 |
| +1 to +30 | Instability | Type | tAI |  |  |  |  |  |  | 0.618097 |
| +1 to +30 | Instability | Type | uAUG |  |  |  |  |  |  | 0.616485 |
| +1 to +30 | Instability | length | tAI |  |  |  |  |  |  | 0.612105 |
| +1 to +30 | Instability | length | uAUG |  |  |  |  |  |  | 0.613426 |
| +1 to +30 | Instability | tAI | uAUG |  |  |  |  |  |  | 0.611296 |
| +1 to +30 | Start | Stop | Type |  |  |  |  |  |  | 0.623745 |
| +1 to +30 | Start | Stop | length |  |  |  |  |  |  | 0.622146 |
| +1 to +30 | Start | Stop | tAI |  |  |  |  |  |  | 0.61827 |
| +1 to +30 | Start | Stop | uAUG |  |  |  |  |  |  | 0.617278 |
| +1 to +30 | Start | Type | length |  |  |  |  |  |  | 0.622794 |
| +1 to +30 | Start | Type | tAI |  |  |  |  |  |  | 0.622688 |
| +1 to +30 | Start | Type | uAUG |  |  |  |  |  |  | 0.621412 |
| +1 to +30 | Start | length | tAI |  |  |  |  |  |  | 0.618162 |
| +1 to +30 | Start | length | uAUG |  |  |  |  |  |  | 0.620501 |
| +1 to +30 | Start | tAI | uAUG |  |  |  |  |  |  | 0.617609 |
| +1 to +30 | Stop | Type | length |  |  |  |  |  |  | 0.624301 |
| +1 to +30 | Stop | Type | tAI |  |  |  |  |  |  | 0.622579 |
| +1 to +30 | Stop | Type | uAUG |  |  |  |  |  |  | 0.620303 |
| +1 to +30 | Stop | length | tAI |  |  |  |  |  |  | 0.619097 |
| +1 to +30 | Stop | length | uAUG |  |  |  |  |  |  | 0.619731 |
| +1 to +30 | Stop | tAI | uAUG |  |  |  |  |  |  | 0.616354 |
| +1 to +30 | Type | length | tAI |  |  |  |  |  |  | 0.62204 |
| +1 to +30 | Type | length | uAUG |  |  |  |  |  |  | 0.621577 |
| +1 to +30 | Type | tAI | uAUG |  |  |  |  |  |  | 0.620248 |
| +1 to +30 | length | tAI | uAUG |  |  |  |  |  |  | 0.615128 |
| CAI | Half-life | Instability | Start |  |  |  |  |  |  | 0.602548 |
| CAI | Half-life | Instability | Stop |  |  |  |  |  |  | 0.602665 |
| CAI | Half-life | Instability | Type |  |  |  |  |  |  | 0.605624 |
| CAI | Half-life | Instability | length |  |  |  |  |  |  | 0.605129 |
| CAI | Half-life | Instability | tAI |  |  |  |  |  |  | 0.599205 |
| CAI | Half-life | Instability | uAUG |  |  |  |  |  |  | 0.599051 |
| CAI | Half-life | Start | Stop |  |  |  |  |  |  | 0.605447 |
| CAI | Half-life | Start | Type |  |  |  |  |  |  | 0.609997 |
| CAI | Half-life | Start | length |  |  |  |  |  |  | 0.613459 |
| CAI | Half-life | Start | tAI |  |  |  |  |  |  | 0.603342 |
| CAI | Half-life | Start | uAUG |  |  |  |  |  |  | 0.601112 |
| CAI | Half-life | Stop | Type |  |  |  |  |  |  | 0.613204 |
| CAI | Half-life | Stop | length |  |  |  |  |  |  | 0.612785 |
| CAI | Half-life | Stop | tAI |  |  |  |  |  |  | 0.603286 |
| CAI | Half-life | Stop | uAUG |  |  |  |  |  |  | 0.601513 |
| CAI | Half-life | Type | length |  |  |  |  |  |  | 0.615163 |
| CAI | Half-life | Type | tAI |  |  |  |  |  |  | 0.607313 |
| CAI | Half-life | Type | uAUG |  |  |  |  |  |  | 0.603358 |
| CAI | Half-life | length | tAI |  |  |  |  |  |  | 0.604219 |
| CAI | Half-life | length | uAUG |  |  |  |  |  |  | 0.608046 |
| CAI | Half-life | tAI | uAUG |  |  |  |  |  |  | 0.596982 |
| CAI | Instability | Start | Stop |  |  |  |  |  |  | 0.606625 |
| CAI | Instability | Start | Type |  |  |  |  |  |  | 0.608862 |
| CAI | Instability | Start | length |  |  |  |  |  |  | 0.609325 |
| CAI | Instability | Start | tAI |  |  |  |  |  |  | 0.605227 |
| CAI | Instability | Start | uAUG |  |  |  |  |  |  | 0.603716 |
| CAI | Instability | Stop | Type |  |  |  |  |  |  | 0.610258 |
| CAI | Instability | Stop | length |  |  |  |  |  |  | 0.610013 |
| CAI | Instability | Stop | tAI |  |  |  |  |  |  | 0.604477 |
| CAI | Instability | Stop | uAUG |  |  |  |  |  |  | 0.60404 |
| CAI | Instability | Type | length |  |  |  |  |  |  | 0.61034 |
| CAI | Instability | Type | tAI |  |  |  |  |  |  | 0.607856 |
| CAI | Instability | Type | uAUG |  |  |  |  |  |  | 0.604344 |
| CAI | Instability | length | tAI |  |  |  |  |  |  | 0.603585 |
| CAI | Instability | length | uAUG |  |  |  |  |  |  | 0.605013 |
| CAI | Instability | tAI | uAUG |  |  |  |  |  |  | 0.598021 |
| CAI | Start | Stop | Type |  |  |  |  |  |  | 0.615812 |
| CAI | Start | Stop | length |  |  |  |  |  |  | 0.616322 |
| CAI | Start | Stop | tAI |  |  |  |  |  |  | 0.61072 |
| CAI | Start | Stop | uAUG |  |  |  |  |  |  | 0.607721 |
| CAI | Start | Type | length |  |  |  |  |  |  | 0.618548 |
| CAI | Start | Type | tAI |  |  |  |  |  |  | 0.610819 |
| CAI | Start | Type | uAUG |  |  |  |  |  |  | 0.611897 |
| CAI | Start | length | tAI |  |  |  |  |  |  | 0.609947 |
| CAI | Start | length | uAUG |  |  |  |  |  |  | 0.613517 |
| CAI | Start | tAI | uAUG |  |  |  |  |  |  | 0.60391 |
| CAI | Stop | Type | length |  |  |  |  |  |  | 0.618819 |
| CAI | Stop | Type | tAI |  |  |  |  |  |  | 0.61423 |
| CAI | Stop | Type | uAUG |  |  |  |  |  |  | 0.611129 |
| CAI | Stop | length | tAI |  |  |  |  |  |  | 0.611516 |
| CAI | Stop | length | uAUG |  |  |  |  |  |  | 0.611506 |
| CAI | Stop | tAI | uAUG |  |  |  |  |  |  | 0.60541 |
| CAI | Type | length | tAI |  |  |  |  |  |  | 0.613109 |
| CAI | Type | length | uAUG |  |  |  |  |  |  | 0.617432 |
| CAI | Type | tAI | uAUG |  |  |  |  |  |  | 0.607954 |
| CAI | length | tAI | uAUG |  |  |  |  |  |  | 0.605881 |
| Half-life | Instability | Start | Stop |  |  |  |  |  |  | 0.614492 |
| Half-life | Instability | Start | Type |  |  |  |  |  |  | 0.616424 |
| Half-life | Instability | Start | length |  |  |  |  |  |  | 0.615947 |
| Half-life | Instability | Start | tAI |  |  |  |  |  |  | 0.613931 |
| Half-life | Instability | Start | uAUG |  |  |  |  |  |  | 0.61128 |
| Half-life | Instability | Stop | Type |  |  |  |  |  |  | 0.617358 |
| Half-life | Instability | Stop | length |  |  |  |  |  |  | 0.615952 |
| Half-life | Instability | Stop | tAI |  |  |  |  |  |  | 0.613357 |
| Half-life | Instability | Stop | uAUG |  |  |  |  |  |  | 0.610754 |
| Half-life | Instability | Type | length |  |  |  |  |  |  | 0.61544 |
| Half-life | Instability | Type | tAI |  |  |  |  |  |  | 0.615981 |
| Half-life | Instability | Type | uAUG |  |  |  |  |  |  | 0.611805 |
| Half-life | Instability | length | tAI |  |  |  |  |  |  | 0.610664 |
| Half-life | Instability | length | uAUG |  |  |  |  |  |  | 0.610996 |
| Half-life | Instability | tAI | uAUG |  |  |  |  |  |  | 0.607084 |
| Half-life | Start | Stop | Type |  |  |  |  |  |  | 0.622514 |
| Half-life | Start | Stop | length |  |  |  |  |  |  | 0.622033 |
| Half-life | Start | Stop | tAI |  |  |  |  |  |  | 0.616858 |
| Half-life | Start | Stop | uAUG |  |  |  |  |  |  | 0.614532 |
| Half-life | Start | Type | length |  |  |  |  |  |  | 0.622221 |
| Half-life | Start | Type | tAI |  |  |  |  |  |  | 0.621451 |
| Half-life | Start | Type | uAUG |  |  |  |  |  |  | 0.620072 |
| Half-life | Start | length | tAI |  |  |  |  |  |  | 0.615798 |
| Half-life | Start | length | uAUG |  |  |  |  |  |  | 0.619207 |
| Half-life | Start | tAI | uAUG |  |  |  |  |  |  | 0.615013 |
| Half-life | Stop | Type | length |  |  |  |  |  |  | 0.623289 |
| Half-life | Stop | Type | tAI |  |  |  |  |  |  | 0.6217 |
| Half-life | Stop | Type | uAUG |  |  |  |  |  |  | 0.617952 |
| Half-life | Stop | length | tAI |  |  |  |  |  |  | 0.618022 |
| Half-life | Stop | length | uAUG |  |  |  |  |  |  | 0.617879 |
| Half-life | Stop | tAI | uAUG |  |  |  |  |  |  | 0.612234 |
| Half-life | Type | length | tAI |  |  |  |  |  |  | 0.619738 |
| Half-life | Type | length | uAUG |  |  |  |  |  |  | 0.618837 |
| Half-life | Type | tAI | uAUG |  |  |  |  |  |  | 0.618035 |
| Half-life | length | tAI | uAUG |  |  |  |  |  |  | 0.611065 |
| Instability | Start | Stop | Type |  |  |  |  |  |  | 0.621626 |
| Instability | Start | Stop | length |  |  |  |  |  |  | 0.619724 |
| Instability | Start | Stop | tAI |  |  |  |  |  |  | 0.614996 |
| Instability | Start | Stop | uAUG |  |  |  |  |  |  | 0.615969 |
| Instability | Start | Type | length |  |  |  |  |  |  | 0.61979 |
| Instability | Start | Type | tAI |  |  |  |  |  |  | 0.618391 |
| Instability | Start | Type | uAUG |  |  |  |  |  |  | 0.616302 |
| Instability | Start | length | tAI |  |  |  |  |  |  | 0.61371 |
| Instability | Start | length | uAUG |  |  |  |  |  |  | 0.616012 |
| Instability | Start | tAI | uAUG |  |  |  |  |  |  | 0.614 |
| Instability | Stop | Type | length |  |  |  |  |  |  | 0.62088 |
| Instability | Stop | Type | tAI |  |  |  |  |  |  | 0.616789 |
| Instability | Stop | Type | uAUG |  |  |  |  |  |  | 0.617803 |
| Instability | Stop | length | tAI |  |  |  |  |  |  | 0.616421 |
| Instability | Stop | length | uAUG |  |  |  |  |  |  | 0.61791 |
| Instability | Stop | tAI | uAUG |  |  |  |  |  |  | 0.611275 |
| Instability | Type | length | tAI |  |  |  |  |  |  | 0.616894 |
| Instability | Type | length | uAUG |  |  |  |  |  |  | 0.616489 |
| Instability | Type | tAI | uAUG |  |  |  |  |  |  | 0.614986 |
| Instability | length | tAI | uAUG |  |  |  |  |  |  | 0.610361 |
| Start | Stop | Type | length |  |  |  |  |  |  | 0.625313 |
| Start | Stop | Type | tAI |  |  |  |  |  |  | 0.623278 |
| Start | Stop | Type | uAUG |  |  |  |  |  |  | 0.62218 |
| Start | Stop | length | tAI |  |  |  |  |  |  | 0.620451 |
| Start | Stop | length | uAUG |  |  |  |  |  |  | 0.621755 |
| Start | Stop | tAI | uAUG |  |  |  |  |  |  | 0.617299 |
| Start | Type | length | tAI |  |  |  |  |  |  | 0.621909 |
| Start | Type | length | uAUG |  |  |  |  |  |  | 0.622328 |
| Start | Type | tAI | uAUG |  |  |  |  |  |  | 0.619961 |
| Start | length | tAI | uAUG |  |  |  |  |  |  | 0.616883 |
| Stop | Type | length | tAI |  |  |  |  |  |  | 0.623969 |
| Stop | Type | length | uAUG |  |  |  |  |  |  | 0.623426 |
| Stop | Type | tAI | uAUG |  |  |  |  |  |  | 0.6201 |
| Stop | length | tAI | uAUG |  |  |  |  |  |  | 0.617257 |
| Type | length | tAI | uAUG |  |  |  |  |  |  | 0.620503 |
| +1 to +30 | CAI | Half-life | Instability | Start |  |  |  |  |  | 0.602723 |
| +1 to +30 | CAI | Half-life | Instability | Stop |  |  |  |  |  | 0.602807 |
| +1 to +30 | CAI | Half-life | Instability | Type |  |  |  |  |  | 0.605008 |
| +1 to +30 | CAI | Half-life | Instability | length |  |  |  |  |  | 0.604426 |
| +1 to +30 | CAI | Half-life | Instability | tAI |  |  |  |  |  | 0.598362 |
| +1 to +30 | CAI | Half-life | Instability | uAUG |  |  |  |  |  | 0.599547 |
| +1 to +30 | CAI | Half-life | Start | Stop |  |  |  |  |  | 0.605115 |
| +1 to +30 | CAI | Half-life | Start | Type |  |  |  |  |  | 0.610052 |
| +1 to +30 | CAI | Half-life | Start | length |  |  |  |  |  | 0.613295 |
| +1 to +30 | CAI | Half-life | Start | tAI |  |  |  |  |  | 0.603957 |
| +1 to +30 | CAI | Half-life | Start | uAUG |  |  |  |  |  | 0.600799 |
| +1 to +30 | CAI | Half-life | Stop | Type |  |  |  |  |  | 0.613262 |
| +1 to +30 | CAI | Half-life | Stop | length |  |  |  |  |  | 0.612957 |
| +1 to +30 | CAI | Half-life | Stop | tAI |  |  |  |  |  | 0.602265 |
| +1 to +30 | CAI | Half-life | Stop | uAUG |  |  |  |  |  | 0.599718 |
| +1 to +30 | CAI | Half-life | Type | length |  |  |  |  |  | 0.614753 |
| +1 to +30 | CAI | Half-life | Type | tAI |  |  |  |  |  | 0.608498 |
| +1 to +30 | CAI | Half-life | Type | uAUG |  |  |  |  |  | 0.604272 |
| +1 to +30 | CAI | Half-life | length | tAI |  |  |  |  |  | 0.604554 |
| +1 to +30 | CAI | Half-life | length | uAUG |  |  |  |  |  | 0.607934 |
| +1 to +30 | CAI | Half-life | tAI | uAUG |  |  |  |  |  | 0.597333 |
| +1 to +30 | CAI | Instability | Start | Stop |  |  |  |  |  | 0.607091 |
| +1 to +30 | CAI | Instability | Start | Type |  |  |  |  |  | 0.609755 |
| +1 to +30 | CAI | Instability | Start | length |  |  |  |  |  | 0.609458 |
| +1 to +30 | CAI | Instability | Start | tAI |  |  |  |  |  | 0.604763 |
| +1 to +30 | CAI | Instability | Start | uAUG |  |  |  |  |  | 0.603373 |
| +1 to +30 | CAI | Instability | Stop | Type |  |  |  |  |  | 0.610469 |
| +1 to +30 | CAI | Instability | Stop | length |  |  |  |  |  | 0.610073 |
| +1 to +30 | CAI | Instability | Stop | tAI |  |  |  |  |  | 0.604507 |
| +1 to +30 | CAI | Instability | Stop | uAUG |  |  |  |  |  | 0.603721 |
| +1 to +30 | CAI | Instability | Type | length |  |  |  |  |  | 0.610693 |
| +1 to +30 | CAI | Instability | Type | tAI |  |  |  |  |  | 0.605878 |
| +1 to +30 | CAI | Instability | Type | uAUG |  |  |  |  |  | 0.60466 |
| +1 to +30 | CAI | Instability | length | tAI |  |  |  |  |  | 0.605846 |
| +1 to +30 | CAI | Instability | length | uAUG |  |  |  |  |  | 0.606071 |
| +1 to +30 | CAI | Instability | tAI | uAUG |  |  |  |  |  | 0.598234 |
| +1 to +30 | CAI | Start | Stop | Type |  |  |  |  |  | 0.61573 |
| +1 to +30 | CAI | Start | Stop | length |  |  |  |  |  | 0.616176 |
| +1 to +30 | CAI | Start | Stop | tAI |  |  |  |  |  | 0.610606 |
| +1 to +30 | CAI | Start | Stop | uAUG |  |  |  |  |  | 0.607218 |
| +1 to +30 | CAI | Start | Type | length |  |  |  |  |  | 0.618669 |
| +1 to +30 | CAI | Start | Type | tAI |  |  |  |  |  | 0.612353 |
| +1 to +30 | CAI | Start | Type | uAUG |  |  |  |  |  | 0.61198 |
| +1 to +30 | CAI | Start | length | tAI |  |  |  |  |  | 0.610777 |
| +1 to +30 | CAI | Start | length | uAUG |  |  |  |  |  | 0.613277 |
| +1 to +30 | CAI | Start | tAI | uAUG |  |  |  |  |  | 0.604746 |
| +1 to +30 | CAI | Stop | Type | length |  |  |  |  |  | 0.619304 |
| +1 to +30 | CAI | Stop | Type | tAI |  |  |  |  |  | 0.614183 |
| +1 to +30 | CAI | Stop | Type | uAUG |  |  |  |  |  | 0.610932 |
| +1 to +30 | CAI | Stop | length | tAI |  |  |  |  |  | 0.612059 |
| +1 to +30 | CAI | Stop | length | uAUG |  |  |  |  |  | 0.611826 |
| +1 to +30 | CAI | Stop | tAI | uAUG |  |  |  |  |  | 0.605566 |
| +1 to +30 | CAI | Type | length | tAI |  |  |  |  |  | 0.61399 |
| +1 to +30 | CAI | Type | length | uAUG |  |  |  |  |  | 0.6178 |
| +1 to +30 | CAI | Type | tAI | uAUG |  |  |  |  |  | 0.607652 |
| +1 to +30 | CAI | length | tAI | uAUG |  |  |  |  |  | 0.607399 |
| +1 to +30 | Half-life | Instability | Start | Stop |  |  |  |  |  | 0.614277 |
| +1 to +30 | Half-life | Instability | Start | Type |  |  |  |  |  | 0.6168 |
| +1 to +30 | Half-life | Instability | Start | length |  |  |  |  |  | 0.616469 |
| +1 to +30 | Half-life | Instability | Start | tAI |  |  |  |  |  | 0.614257 |
| +1 to +30 | Half-life | Instability | Start | uAUG |  |  |  |  |  | 0.610986 |
| +1 to +30 | Half-life | Instability | Stop | Type |  |  |  |  |  | 0.617666 |
| +1 to +30 | Half-life | Instability | Stop | length |  |  |  |  |  | 0.616098 |
| +1 to +30 | Half-life | Instability | Stop | tAI |  |  |  |  |  | 0.612472 |
| +1 to +30 | Half-life | Instability | Stop | uAUG |  |  |  |  |  | 0.611081 |
| +1 to +30 | Half-life | Instability | Type | length |  |  |  |  |  | 0.615655 |
| +1 to +30 | Half-life | Instability | Type | tAI |  |  |  |  |  | 0.617059 |
| +1 to +30 | Half-life | Instability | Type | uAUG |  |  |  |  |  | 0.61259 |
| +1 to +30 | Half-life | Instability | length | tAI |  |  |  |  |  | 0.611031 |
| +1 to +30 | Half-life | Instability | length | uAUG |  |  |  |  |  | 0.611974 |
| +1 to +30 | Half-life | Instability | tAI | uAUG |  |  |  |  |  | 0.607866 |
| +1 to +30 | Half-life | Start | Stop | Type |  |  |  |  |  | 0.622119 |
| +1 to +30 | Half-life | Start | Stop | length |  |  |  |  |  | 0.6223 |
| +1 to +30 | Half-life | Start | Stop | tAI |  |  |  |  |  | 0.616295 |
| +1 to +30 | Half-life | Start | Stop | uAUG |  |  |  |  |  | 0.61326 |
| +1 to +30 | Half-life | Start | Type | length |  |  |  |  |  | 0.62199 |
| +1 to +30 | Half-life | Start | Type | tAI |  |  |  |  |  | 0.621504 |
| +1 to +30 | Half-life | Start | Type | uAUG |  |  |  |  |  | 0.619717 |
| +1 to +30 | Half-life | Start | length | tAI |  |  |  |  |  | 0.615906 |
| +1 to +30 | Half-life | Start | length | uAUG |  |  |  |  |  | 0.619423 |
| +1 to +30 | Half-life | Start | tAI | uAUG |  |  |  |  |  | 0.615055 |
| +1 to +30 | Half-life | Stop | Type | length |  |  |  |  |  | 0.623191 |
| +1 to +30 | Half-life | Stop | Type | tAI |  |  |  |  |  | 0.621402 |
| +1 to +30 | Half-life | Stop | Type | uAUG |  |  |  |  |  | 0.617421 |
| +1 to +30 | Half-life | Stop | length | tAI |  |  |  |  |  | 0.617913 |
| +1 to +30 | Half-life | Stop | length | uAUG |  |  |  |  |  | 0.616795 |
| +1 to +30 | Half-life | Stop | tAI | uAUG |  |  |  |  |  | 0.612073 |
| +1 to +30 | Half-life | Type | length | tAI |  |  |  |  |  | 0.620261 |
| +1 to +30 | Half-life | Type | length | uAUG |  |  |  |  |  | 0.619205 |
| +1 to +30 | Half-life | Type | tAI | uAUG |  |  |  |  |  | 0.618127 |
| +1 to +30 | Half-life | length | tAI | uAUG |  |  |  |  |  | 0.611694 |
| +1 to +30 | Instability | Start | Stop | Type |  |  |  |  |  | 0.621381 |
| +1 to +30 | Instability | Start | Stop | length |  |  |  |  |  | 0.619744 |
| +1 to +30 | Instability | Start | Stop | tAI |  |  |  |  |  | 0.615185 |
| +1 to +30 | Instability | Start | Stop | uAUG |  |  |  |  |  | 0.616024 |
| +1 to +30 | Instability | Start | Type | length |  |  |  |  |  | 0.620087 |
| +1 to +30 | Instability | Start | Type | tAI |  |  |  |  |  | 0.618429 |
| +1 to +30 | Instability | Start | Type | uAUG |  |  |  |  |  | 0.616603 |
| +1 to +30 | Instability | Start | length | tAI |  |  |  |  |  | 0.613607 |
| +1 to +30 | Instability | Start | length | uAUG |  |  |  |  |  | 0.617104 |
| +1 to +30 | Instability | Start | tAI | uAUG |  |  |  |  |  | 0.613753 |
| +1 to +30 | Instability | Stop | Type | length |  |  |  |  |  | 0.620716 |
| +1 to +30 | Instability | Stop | Type | tAI |  |  |  |  |  | 0.616654 |
| +1 to +30 | Instability | Stop | Type | uAUG |  |  |  |  |  | 0.61765 |
| +1 to +30 | Instability | Stop | length | tAI |  |  |  |  |  | 0.616196 |
| +1 to +30 | Instability | Stop | length | uAUG |  |  |  |  |  | 0.617618 |
| +1 to +30 | Instability | Stop | tAI | uAUG |  |  |  |  |  | 0.611189 |
| +1 to +30 | Instability | Type | length | tAI |  |  |  |  |  | 0.617056 |
| +1 to +30 | Instability | Type | length | uAUG |  |  |  |  |  | 0.616856 |
| +1 to +30 | Instability | Type | tAI | uAUG |  |  |  |  |  | 0.615097 |
| +1 to +30 | Instability | length | tAI | uAUG |  |  |  |  |  | 0.610356 |
| +1 to +30 | Start | Stop | Type | length |  |  |  |  |  | 0.625104 |
| +1 to +30 | Start | Stop | Type | tAI |  |  |  |  |  | 0.623072 |
| +1 to +30 | Start | Stop | Type | uAUG |  |  |  |  |  | 0.622062 |
| +1 to +30 | Start | Stop | length | tAI |  |  |  |  |  | 0.620755 |
| +1 to +30 | Start | Stop | length | uAUG |  |  |  |  |  | 0.62128 |
| +1 to +30 | Start | Stop | tAI | uAUG |  |  |  |  |  | 0.61735 |
| +1 to +30 | Start | Type | length | tAI |  |  |  |  |  | 0.621983 |
| +1 to +30 | Start | Type | length | uAUG |  |  |  |  |  | 0.622645 |
| +1 to +30 | Start | Type | tAI | uAUG |  |  |  |  |  | 0.620028 |
| +1 to +30 | Start | length | tAI | uAUG |  |  |  |  |  | 0.617404 |
| +1 to +30 | Stop | Type | length | tAI |  |  |  |  |  | 0.623196 |
| +1 to +30 | Stop | Type | length | uAUG |  |  |  |  |  | 0.623153 |
| +1 to +30 | Stop | Type | tAI | uAUG |  |  |  |  |  | 0.620207 |
| +1 to +30 | Stop | length | tAI | uAUG |  |  |  |  |  | 0.617225 |
| +1 to +30 | Type | length | tAI | uAUG |  |  |  |  |  | 0.621273 |
| CAI | Half-life | Instability | Start | Stop |  |  |  |  |  | 0.603834 |
| CAI | Half-life | Instability | Start | Type |  |  |  |  |  | 0.606625 |
| CAI | Half-life | Instability | Start | length |  |  |  |  |  | 0.608247 |
| CAI | Half-life | Instability | Start | tAI |  |  |  |  |  | 0.602712 |
| CAI | Half-life | Instability | Start | uAUG |  |  |  |  |  | 0.600344 |
| CAI | Half-life | Instability | Stop | Type |  |  |  |  |  | 0.607241 |
| CAI | Half-life | Instability | Stop | length |  |  |  |  |  | 0.609156 |
| CAI | Half-life | Instability | Stop | tAI |  |  |  |  |  | 0.603222 |
| CAI | Half-life | Instability | Stop | uAUG |  |  |  |  |  | 0.598051 |
| CAI | Half-life | Instability | Type | length |  |  |  |  |  | 0.607535 |
| CAI | Half-life | Instability | Type | tAI |  |  |  |  |  | 0.60453 |
| CAI | Half-life | Instability | Type | uAUG |  |  |  |  |  | 0.600636 |
| CAI | Half-life | Instability | length | tAI |  |  |  |  |  | 0.599955 |
| CAI | Half-life | Instability | length | uAUG |  |  |  |  |  | 0.603032 |
| CAI | Half-life | Instability | tAI | uAUG |  |  |  |  |  | 0.596191 |
| CAI | Half-life | Start | Stop | Type |  |  |  |  |  | 0.614997 |
| CAI | Half-life | Start | Stop | length |  |  |  |  |  | 0.613871 |
| CAI | Half-life | Start | Stop | tAI |  |  |  |  |  | 0.603968 |
| CAI | Half-life | Start | Stop | uAUG |  |  |  |  |  | 0.603287 |
| CAI | Half-life | Start | Type | length |  |  |  |  |  | 0.616994 |
| CAI | Half-life | Start | Type | tAI |  |  |  |  |  | 0.606961 |
| CAI | Half-life | Start | Type | uAUG |  |  |  |  |  | 0.606927 |
| CAI | Half-life | Start | length | tAI |  |  |  |  |  | 0.608874 |
| CAI | Half-life | Start | length | uAUG |  |  |  |  |  | 0.610845 |
| CAI | Half-life | Start | tAI | uAUG |  |  |  |  |  | 0.599708 |
| CAI | Half-life | Stop | Type | length |  |  |  |  |  | 0.617528 |
| CAI | Half-life | Stop | Type | tAI |  |  |  |  |  | 0.61116 |
| CAI | Half-life | Stop | Type | uAUG |  |  |  |  |  | 0.608482 |
| CAI | Half-life | Stop | length | tAI |  |  |  |  |  | 0.610361 |
| CAI | Half-life | Stop | length | uAUG |  |  |  |  |  | 0.609943 |
| CAI | Half-life | Stop | tAI | uAUG |  |  |  |  |  | 0.6005 |
| CAI | Half-life | Type | length | tAI |  |  |  |  |  | 0.611362 |
| CAI | Half-life | Type | length | uAUG |  |  |  |  |  | 0.6134 |
| CAI | Half-life | Type | tAI | uAUG |  |  |  |  |  | 0.605075 |
| CAI | Half-life | length | tAI | uAUG |  |  |  |  |  | 0.602905 |
| CAI | Instability | Start | Stop | Type |  |  |  |  |  | 0.612987 |
| CAI | Instability | Start | Stop | length |  |  |  |  |  | 0.61154 |
| CAI | Instability | Start | Stop | tAI |  |  |  |  |  | 0.606806 |
| CAI | Instability | Start | Stop | uAUG |  |  |  |  |  | 0.60421 |
| CAI | Instability | Start | Type | length |  |  |  |  |  | 0.615562 |
| CAI | Instability | Start | Type | tAI |  |  |  |  |  | 0.607996 |
| CAI | Instability | Start | Type | uAUG |  |  |  |  |  | 0.6065 |
| CAI | Instability | Start | length | tAI |  |  |  |  |  | 0.608554 |
| CAI | Instability | Start | length | uAUG |  |  |  |  |  | 0.608559 |
| CAI | Instability | Start | tAI | uAUG |  |  |  |  |  | 0.602462 |
| CAI | Instability | Stop | Type | length |  |  |  |  |  | 0.615118 |
| CAI | Instability | Stop | Type | tAI |  |  |  |  |  | 0.608346 |
| CAI | Instability | Stop | Type | uAUG |  |  |  |  |  | 0.605924 |
| CAI | Instability | Stop | length | tAI |  |  |  |  |  | 0.608667 |
| CAI | Instability | Stop | length | uAUG |  |  |  |  |  | 0.608139 |
| CAI | Instability | Stop | tAI | uAUG |  |  |  |  |  | 0.600449 |
| CAI | Instability | Type | length | tAI |  |  |  |  |  | 0.610028 |
| CAI | Instability | Type | length | uAUG |  |  |  |  |  | 0.610138 |
| CAI | Instability | Type | tAI | uAUG |  |  |  |  |  | 0.605822 |
| CAI | Instability | length | tAI | uAUG |  |  |  |  |  | 0.602216 |
| CAI | Start | Stop | Type | length |  |  |  |  |  | 0.619982 |
| CAI | Start | Stop | Type | tAI |  |  |  |  |  | 0.614396 |
| CAI | Start | Stop | Type | uAUG |  |  |  |  |  | 0.611589 |
| CAI | Start | Stop | length | tAI |  |  |  |  |  | 0.612547 |
| CAI | Start | Stop | length | uAUG |  |  |  |  |  | 0.614735 |
| CAI | Start | Stop | tAI | uAUG |  |  |  |  |  | 0.607327 |
| CAI | Start | Type | length | tAI |  |  |  |  |  | 0.615585 |
| CAI | Start | Type | length | uAUG |  |  |  |  |  | 0.617725 |
| CAI | Start | Type | tAI | uAUG |  |  |  |  |  | 0.607893 |
| CAI | Start | length | tAI | uAUG |  |  |  |  |  | 0.609216 |
| CAI | Stop | Type | length | tAI |  |  |  |  |  | 0.618212 |
| CAI | Stop | Type | length | uAUG |  |  |  |  |  | 0.616024 |
| CAI | Stop | Type | tAI | uAUG |  |  |  |  |  | 0.610638 |
| CAI | Stop | length | tAI | uAUG |  |  |  |  |  | 0.610276 |
| CAI | Type | length | tAI | uAUG |  |  |  |  |  | 0.611212 |
| Half-life | Instability | Start | Stop | Type |  |  |  |  |  | 0.618969 |
| Half-life | Instability | Start | Stop | length |  |  |  |  |  | 0.619723 |
| Half-life | Instability | Start | Stop | tAI |  |  |  |  |  | 0.613702 |
| Half-life | Instability | Start | Stop | uAUG |  |  |  |  |  | 0.611622 |
| Half-life | Instability | Start | Type | length |  |  |  |  |  | 0.618681 |
| Half-life | Instability | Start | Type | tAI |  |  |  |  |  | 0.616621 |
| Half-life | Instability | Start | Type | uAUG |  |  |  |  |  | 0.61397 |
| Half-life | Instability | Start | length | tAI |  |  |  |  |  | 0.612153 |
| Half-life | Instability | Start | length | uAUG |  |  |  |  |  | 0.614168 |
| Half-life | Instability | Start | tAI | uAUG |  |  |  |  |  | 0.610977 |
| Half-life | Instability | Stop | Type | length |  |  |  |  |  | 0.619256 |
| Half-life | Instability | Stop | Type | tAI |  |  |  |  |  | 0.617694 |
| Half-life | Instability | Stop | Type | uAUG |  |  |  |  |  | 0.61578 |
| Half-life | Instability | Stop | length | tAI |  |  |  |  |  | 0.614565 |
| Half-life | Instability | Stop | length | uAUG |  |  |  |  |  | 0.613793 |
| Half-life | Instability | Stop | tAI | uAUG |  |  |  |  |  | 0.610814 |
| Half-life | Instability | Type | length | tAI |  |  |  |  |  | 0.614503 |
| Half-life | Instability | Type | length | uAUG |  |  |  |  |  | 0.612949 |
| Half-life | Instability | Type | tAI | uAUG |  |  |  |  |  | 0.612013 |
| Half-life | Instability | length | tAI | uAUG |  |  |  |  |  | 0.60862 |
| Half-life | Start | Stop | Type | length |  |  |  |  |  | 0.62565 |
| Half-life | Start | Stop | Type | tAI |  |  |  |  |  | 0.623013 |
| Half-life | Start | Stop | Type | uAUG |  |  |  |  |  | 0.62066 |
| Half-life | Start | Stop | length | tAI |  |  |  |  |  | 0.618208 |
| Half-life | Start | Stop | length | uAUG |  |  |  |  |  | 0.621036 |
| Half-life | Start | Stop | tAI | uAUG |  |  |  |  |  | 0.615021 |
| Half-life | Start | Type | length | tAI |  |  |  |  |  | 0.620389 |
| Half-life | Start | Type | length | uAUG |  |  |  |  |  | 0.620286 |
| Half-life | Start | Type | tAI | uAUG |  |  |  |  |  | 0.619197 |
| Half-life | Start | length | tAI | uAUG |  |  |  |  |  | 0.613982 |
| Half-life | Stop | Type | length | tAI |  |  |  |  |  | 0.622388 |
| Half-life | Stop | Type | length | uAUG |  |  |  |  |  | 0.621411 |
| Half-life | Stop | Type | tAI | uAUG |  |  |  |  |  | 0.618392 |
| Half-life | Stop | length | tAI | uAUG |  |  |  |  |  | 0.61635 |
| Half-life | Type | length | tAI | uAUG |  |  |  |  |  | 0.618498 |
| Instability | Start | Stop | Type | length |  |  |  |  |  | 0.623271 |
| Instability | Start | Stop | Type | tAI |  |  |  |  |  | 0.618283 |
| Instability | Start | Stop | Type | uAUG |  |  |  |  |  | 0.620557 |
| Instability | Start | Stop | length | tAI |  |  |  |  |  | 0.61612 |
| Instability | Start | Stop | length | uAUG |  |  |  |  |  | 0.62037 |
| Instability | Start | Stop | tAI | uAUG |  |  |  |  |  | 0.61395 |
| Instability | Start | Type | length | tAI |  |  |  |  |  | 0.618014 |
| Instability | Start | Type | length | uAUG |  |  |  |  |  | 0.619381 |
| Instability | Start | Type | tAI | uAUG |  |  |  |  |  | 0.616401 |
| Instability | Start | length | tAI | uAUG |  |  |  |  |  | 0.611974 |
| Instability | Stop | Type | length | tAI |  |  |  |  |  | 0.619592 |
| Instability | Stop | Type | length | uAUG |  |  |  |  |  | 0.62055 |
| Instability | Stop | Type | tAI | uAUG |  |  |  |  |  | 0.614235 |
| Instability | Stop | length | tAI | uAUG |  |  |  |  |  | 0.614318 |
| Instability | Type | length | tAI | uAUG |  |  |  |  |  | 0.616351 |
| Start | Stop | Type | length | tAI |  |  |  |  |  | 0.623615 |
| Start | Stop | Type | length | uAUG |  |  |  |  |  | 0.624892 |
| Start | Stop | Type | tAI | uAUG |  |  |  |  |  | 0.621299 |
| Start | Stop | length | tAI | uAUG |  |  |  |  |  | 0.619183 |
| Start | Type | length | tAI | uAUG |  |  |  |  |  | 0.620432 |
| Stop | Type | length | tAI | uAUG |  |  |  |  |  | 0.62176 |
| +1 to +30 | CAI | Half-life | Instability | Start | Stop |  |  |  |  | 0.604325 |
| +1 to +30 | CAI | Half-life | Instability | Start | Type |  |  |  |  | 0.607449 |
| +1 to +30 | CAI | Half-life | Instability | Start | length |  |  |  |  | 0.608884 |
| +1 to +30 | CAI | Half-life | Instability | Start | tAI |  |  |  |  | 0.601933 |
| +1 to +30 | CAI | Half-life | Instability | Start | uAUG |  |  |  |  | 0.600738 |
| +1 to +30 | CAI | Half-life | Instability | Stop | Type |  |  |  |  | 0.607842 |
| +1 to +30 | CAI | Half-life | Instability | Stop | length |  |  |  |  | 0.609709 |
| +1 to +30 | CAI | Half-life | Instability | Stop | tAI |  |  |  |  | 0.60412 |
| +1 to +30 | CAI | Half-life | Instability | Stop | uAUG |  |  |  |  | 0.598779 |
| +1 to +30 | CAI | Half-life | Instability | Type | length |  |  |  |  | 0.607234 |
| +1 to +30 | CAI | Half-life | Instability | Type | tAI |  |  |  |  | 0.604773 |
| +1 to +30 | CAI | Half-life | Instability | Type | uAUG |  |  |  |  | 0.600598 |
| +1 to +30 | CAI | Half-life | Instability | length | tAI |  |  |  |  | 0.601762 |
| +1 to +30 | CAI | Half-life | Instability | length | uAUG |  |  |  |  | 0.602967 |
| +1 to +30 | CAI | Half-life | Instability | tAI | uAUG |  |  |  |  | 0.596274 |
| +1 to +30 | CAI | Half-life | Start | Stop | Type |  |  |  |  | 0.614736 |
| +1 to +30 | CAI | Half-life | Start | Stop | length |  |  |  |  | 0.613814 |
| +1 to +30 | CAI | Half-life | Start | Stop | tAI |  |  |  |  | 0.604558 |
| +1 to +30 | CAI | Half-life | Start | Stop | uAUG |  |  |  |  | 0.601219 |
| +1 to +30 | CAI | Half-life | Start | Type | length |  |  |  |  | 0.617125 |
| +1 to +30 | CAI | Half-life | Start | Type | tAI |  |  |  |  | 0.608171 |
| +1 to +30 | CAI | Half-life | Start | Type | uAUG |  |  |  |  | 0.60714 |
| +1 to +30 | CAI | Half-life | Start | length | tAI |  |  |  |  | 0.608298 |
| +1 to +30 | CAI | Half-life | Start | length | uAUG |  |  |  |  | 0.612211 |
| +1 to +30 | CAI | Half-life | Start | tAI | uAUG |  |  |  |  | 0.600213 |
| +1 to +30 | CAI | Half-life | Stop | Type | length |  |  |  |  | 0.617108 |
| +1 to +30 | CAI | Half-life | Stop | Type | tAI |  |  |  |  | 0.611094 |
| +1 to +30 | CAI | Half-life | Stop | Type | uAUG |  |  |  |  | 0.609107 |
| +1 to +30 | CAI | Half-life | Stop | length | tAI |  |  |  |  | 0.609823 |
| +1 to +30 | CAI | Half-life | Stop | length | uAUG |  |  |  |  | 0.60988 |
| +1 to +30 | CAI | Half-life | Stop | tAI | uAUG |  |  |  |  | 0.600478 |
| +1 to +30 | CAI | Half-life | Type | length | tAI |  |  |  |  | 0.611105 |
| +1 to +30 | CAI | Half-life | Type | length | uAUG |  |  |  |  | 0.613074 |
| +1 to +30 | CAI | Half-life | Type | tAI | uAUG |  |  |  |  | 0.605584 |
| +1 to +30 | CAI | Half-life | length | tAI | uAUG |  |  |  |  | 0.602295 |
| +1 to +30 | CAI | Instability | Start | Stop | Type |  |  |  |  | 0.612941 |
| +1 to +30 | CAI | Instability | Start | Stop | length |  |  |  |  | 0.613366 |
| +1 to +30 | CAI | Instability | Start | Stop | tAI |  |  |  |  | 0.606166 |
| +1 to +30 | CAI | Instability | Start | Stop | uAUG |  |  |  |  | 0.604157 |
| +1 to +30 | CAI | Instability | Start | Type | length |  |  |  |  | 0.616087 |
| +1 to +30 | CAI | Instability | Start | Type | tAI |  |  |  |  | 0.608066 |
| +1 to +30 | CAI | Instability | Start | Type | uAUG |  |  |  |  | 0.607268 |
| +1 to +30 | CAI | Instability | Start | length | tAI |  |  |  |  | 0.60915 |
| +1 to +30 | CAI | Instability | Start | length | uAUG |  |  |  |  | 0.608823 |
| +1 to +30 | CAI | Instability | Start | tAI | uAUG |  |  |  |  | 0.602312 |
| +1 to +30 | CAI | Instability | Stop | Type | length |  |  |  |  | 0.614697 |
| +1 to +30 | CAI | Instability | Stop | Type | tAI |  |  |  |  | 0.60769 |
| +1 to +30 | CAI | Instability | Stop | Type | uAUG |  |  |  |  | 0.605567 |
| +1 to +30 | CAI | Instability | Stop | length | tAI |  |  |  |  | 0.608797 |
| +1 to +30 | CAI | Instability | Stop | length | uAUG |  |  |  |  | 0.609949 |
| +1 to +30 | CAI | Instability | Stop | tAI | uAUG |  |  |  |  | 0.600685 |
| +1 to +30 | CAI | Instability | Type | length | tAI |  |  |  |  | 0.61047 |
| +1 to +30 | CAI | Instability | Type | length | uAUG |  |  |  |  | 0.60991 |
| +1 to +30 | CAI | Instability | Type | tAI | uAUG |  |  |  |  | 0.60554 |
| +1 to +30 | CAI | Instability | length | tAI | uAUG |  |  |  |  | 0.604433 |
| +1 to +30 | CAI | Start | Stop | Type | length |  |  |  |  | 0.619849 |
| +1 to +30 | CAI | Start | Stop | Type | tAI |  |  |  |  | 0.614401 |
| +1 to +30 | CAI | Start | Stop | Type | uAUG |  |  |  |  | 0.611767 |
| +1 to +30 | CAI | Start | Stop | length | tAI |  |  |  |  | 0.612491 |
| +1 to +30 | CAI | Start | Stop | length | uAUG |  |  |  |  | 0.614695 |
| +1 to +30 | CAI | Start | Stop | tAI | uAUG |  |  |  |  | 0.607407 |
| +1 to +30 | CAI | Start | Type | length | tAI |  |  |  |  | 0.616278 |
| +1 to +30 | CAI | Start | Type | length | uAUG |  |  |  |  | 0.617333 |
| +1 to +30 | CAI | Start | Type | tAI | uAUG |  |  |  |  | 0.608174 |
| +1 to +30 | CAI | Start | length | tAI | uAUG |  |  |  |  | 0.609044 |
| +1 to +30 | CAI | Stop | Type | length | tAI |  |  |  |  | 0.618428 |
| +1 to +30 | CAI | Stop | Type | length | uAUG |  |  |  |  | 0.61559 |
| +1 to +30 | CAI | Stop | Type | tAI | uAUG |  |  |  |  | 0.611499 |
| +1 to +30 | CAI | Stop | length | tAI | uAUG |  |  |  |  | 0.611052 |
| +1 to +30 | CAI | Type | length | tAI | uAUG |  |  |  |  | 0.614161 |
| +1 to +30 | Half-life | Instability | Start | Stop | Type |  |  |  |  | 0.618356 |
| +1 to +30 | Half-life | Instability | Start | Stop | length |  |  |  |  | 0.620176 |
| +1 to +30 | Half-life | Instability | Start | Stop | tAI |  |  |  |  | 0.613637 |
| +1 to +30 | Half-life | Instability | Start | Stop | uAUG |  |  |  |  | 0.611704 |
| +1 to +30 | Half-life | Instability | Start | Type | length |  |  |  |  | 0.618795 |
| +1 to +30 | Half-life | Instability | Start | Type | tAI |  |  |  |  | 0.617112 |
| +1 to +30 | Half-life | Instability | Start | Type | uAUG |  |  |  |  | 0.613944 |
| +1 to +30 | Half-life | Instability | Start | length | tAI |  |  |  |  | 0.612679 |
| +1 to +30 | Half-life | Instability | Start | length | uAUG |  |  |  |  | 0.614292 |
| +1 to +30 | Half-life | Instability | Start | tAI | uAUG |  |  |  |  | 0.611395 |
| +1 to +30 | Half-life | Instability | Stop | Type | length |  |  |  |  | 0.619072 |
| +1 to +30 | Half-life | Instability | Stop | Type | tAI |  |  |  |  | 0.617794 |
| +1 to +30 | Half-life | Instability | Stop | Type | uAUG |  |  |  |  | 0.615145 |
| +1 to +30 | Half-life | Instability | Stop | length | tAI |  |  |  |  | 0.614655 |
| +1 to +30 | Half-life | Instability | Stop | length | uAUG |  |  |  |  | 0.613993 |
| +1 to +30 | Half-life | Instability | Stop | tAI | uAUG |  |  |  |  | 0.610326 |
| +1 to +30 | Half-life | Instability | Type | length | tAI |  |  |  |  | 0.615264 |
| +1 to +30 | Half-life | Instability | Type | length | uAUG |  |  |  |  | 0.613115 |
| +1 to +30 | Half-life | Instability | Type | tAI | uAUG |  |  |  |  | 0.611685 |
| +1 to +30 | Half-life | Instability | length | tAI | uAUG |  |  |  |  | 0.609343 |
| +1 to +30 | Half-life | Start | Stop | Type | length |  |  |  |  | 0.626 |
| +1 to +30 | Half-life | Start | Stop | Type | tAI |  |  |  |  | 0.623283 |
| +1 to +30 | Half-life | Start | Stop | Type | uAUG |  |  |  |  | 0.619068 |
| +1 to +30 | Half-life | Start | Stop | length | tAI |  |  |  |  | 0.617725 |
| +1 to +30 | Half-life | Start | Stop | length | uAUG |  |  |  |  | 0.621081 |
| +1 to +30 | Half-life | Start | Stop | tAI | uAUG |  |  |  |  | 0.614613 |
| +1 to +30 | Half-life | Start | Type | length | tAI |  |  |  |  | 0.620537 |
| +1 to +30 | Half-life | Start | Type | length | uAUG |  |  |  |  | 0.620068 |
| +1 to +30 | Half-life | Start | Type | tAI | uAUG |  |  |  |  | 0.619252 |
| +1 to +30 | Half-life | Start | length | tAI | uAUG |  |  |  |  | 0.615232 |
| +1 to +30 | Half-life | Stop | Type | length | tAI |  |  |  |  | 0.621408 |
| +1 to +30 | Half-life | Stop | Type | length | uAUG |  |  |  |  | 0.621665 |
| +1 to +30 | Half-life | Stop | Type | tAI | uAUG |  |  |  |  | 0.618572 |
| +1 to +30 | Half-life | Stop | length | tAI | uAUG |  |  |  |  | 0.616494 |
| +1 to +30 | Half-life | Type | length | tAI | uAUG |  |  |  |  | 0.618688 |
| +1 to +30 | Instability | Start | Stop | Type | length |  |  |  |  | 0.62403 |
| +1 to +30 | Instability | Start | Stop | Type | tAI |  |  |  |  | 0.618093 |
| +1 to +30 | Instability | Start | Stop | Type | uAUG |  |  |  |  | 0.620312 |
| +1 to +30 | Instability | Start | Stop | length | tAI |  |  |  |  | 0.616665 |
| +1 to +30 | Instability | Start | Stop | length | uAUG |  |  |  |  | 0.620427 |
| +1 to +30 | Instability | Start | Stop | tAI | uAUG |  |  |  |  | 0.613627 |
| +1 to +30 | Instability | Start | Type | length | tAI |  |  |  |  | 0.617689 |
| +1 to +30 | Instability | Start | Type | length | uAUG |  |  |  |  | 0.618905 |
| +1 to +30 | Instability | Start | Type | tAI | uAUG |  |  |  |  | 0.616101 |
| +1 to +30 | Instability | Start | length | tAI | uAUG |  |  |  |  | 0.61282 |
| +1 to +30 | Instability | Stop | Type | length | tAI |  |  |  |  | 0.619761 |
| +1 to +30 | Instability | Stop | Type | length | uAUG |  |  |  |  | 0.620478 |
| +1 to +30 | Instability | Stop | Type | tAI | uAUG |  |  |  |  | 0.614379 |
| +1 to +30 | Instability | Stop | length | tAI | uAUG |  |  |  |  | 0.615021 |
| +1 to +30 | Instability | Type | length | tAI | uAUG |  |  |  |  | 0.615729 |
| +1 to +30 | Start | Stop | Type | length | tAI |  |  |  |  | 0.622958 |
| +1 to +30 | Start | Stop | Type | length | uAUG |  |  |  |  | 0.624575 |
| +1 to +30 | Start | Stop | Type | tAI | uAUG |  |  |  |  | 0.620984 |
| +1 to +30 | Start | Stop | length | tAI | uAUG |  |  |  |  | 0.619672 |
| +1 to +30 | Start | Type | length | tAI | uAUG |  |  |  |  | 0.6202 |
| +1 to +30 | Stop | Type | length | tAI | uAUG |  |  |  |  | 0.621548 |
| CAI | Half-life | Instability | Start | Stop | Type |  |  |  |  | 0.608381 |
| CAI | Half-life | Instability | Start | Stop | length |  |  |  |  | 0.610164 |
| CAI | Half-life | Instability | Start | Stop | tAI |  |  |  |  | 0.603349 |
| CAI | Half-life | Instability | Start | Stop | uAUG |  |  |  |  | 0.600494 |
| CAI | Half-life | Instability | Start | Type | length |  |  |  |  | 0.610914 |
| CAI | Half-life | Instability | Start | Type | tAI |  |  |  |  | 0.604623 |
| CAI | Half-life | Instability | Start | Type | uAUG |  |  |  |  | 0.602964 |
| CAI | Half-life | Instability | Start | length | tAI |  |  |  |  | 0.605087 |
| CAI | Half-life | Instability | Start | length | uAUG |  |  |  |  | 0.606259 |
| CAI | Half-life | Instability | Start | tAI | uAUG |  |  |  |  | 0.600702 |
| CAI | Half-life | Instability | Stop | Type | length |  |  |  |  | 0.612714 |
| CAI | Half-life | Instability | Stop | Type | tAI |  |  |  |  | 0.605545 |
| CAI | Half-life | Instability | Stop | Type | uAUG |  |  |  |  | 0.603426 |
| CAI | Half-life | Instability | Stop | length | tAI |  |  |  |  | 0.604931 |
| CAI | Half-life | Instability | Stop | length | uAUG |  |  |  |  | 0.607035 |
| CAI | Half-life | Instability | Stop | tAI | uAUG |  |  |  |  | 0.600655 |
| CAI | Half-life | Instability | Type | length | tAI |  |  |  |  | 0.605967 |
| CAI | Half-life | Instability | Type | length | uAUG |  |  |  |  | 0.605298 |
| CAI | Half-life | Instability | Type | tAI | uAUG |  |  |  |  | 0.602627 |
| CAI | Half-life | Instability | length | tAI | uAUG |  |  |  |  | 0.599371 |
| CAI | Half-life | Start | Stop | Type | length |  |  |  |  | 0.6183 |
| CAI | Half-life | Start | Stop | Type | tAI |  |  |  |  | 0.610084 |
| CAI | Half-life | Start | Stop | Type | uAUG |  |  |  |  | 0.612403 |
| CAI | Half-life | Start | Stop | length | tAI |  |  |  |  | 0.610218 |
| CAI | Half-life | Start | Stop | length | uAUG |  |  |  |  | 0.612023 |
| CAI | Half-life | Start | Stop | tAI | uAUG |  |  |  |  | 0.602287 |
| CAI | Half-life | Start | Type | length | tAI |  |  |  |  | 0.613204 |
| CAI | Half-life | Start | Type | length | uAUG |  |  |  |  | 0.615757 |
| CAI | Half-life | Start | Type | tAI | uAUG |  |  |  |  | 0.604996 |
| CAI | Half-life | Start | length | tAI | uAUG |  |  |  |  | 0.607139 |
| CAI | Half-life | Stop | Type | length | tAI |  |  |  |  | 0.614299 |
| CAI | Half-life | Stop | Type | length | uAUG |  |  |  |  | 0.614044 |
| CAI | Half-life | Stop | Type | tAI | uAUG |  |  |  |  | 0.606751 |
| CAI | Half-life | Stop | length | tAI | uAUG |  |  |  |  | 0.606462 |
| CAI | Half-life | Type | length | tAI | uAUG |  |  |  |  | 0.607209 |
| CAI | Instability | Start | Stop | Type | length |  |  |  |  | 0.61618 |
| CAI | Instability | Start | Stop | Type | tAI |  |  |  |  | 0.60792 |
| CAI | Instability | Start | Stop | Type | uAUG |  |  |  |  | 0.607957 |
| CAI | Instability | Start | Stop | length | tAI |  |  |  |  | 0.609929 |
| CAI | Instability | Start | Stop | length | uAUG |  |  |  |  | 0.611095 |
| CAI | Instability | Start | Stop | tAI | uAUG |  |  |  |  | 0.603708 |
| CAI | Instability | Start | Type | length | tAI |  |  |  |  | 0.610977 |
| CAI | Instability | Start | Type | length | uAUG |  |  |  |  | 0.612471 |
| CAI | Instability | Start | Type | tAI | uAUG |  |  |  |  | 0.605217 |
| CAI | Instability | Start | length | tAI | uAUG |  |  |  |  | 0.605622 |
| CAI | Instability | Stop | Type | length | tAI |  |  |  |  | 0.613389 |
| CAI | Instability | Stop | Type | length | uAUG |  |  |  |  | 0.611009 |
| CAI | Instability | Stop | Type | tAI | uAUG |  |  |  |  | 0.604967 |
| CAI | Instability | Stop | length | tAI | uAUG |  |  |  |  | 0.60532 |
| CAI | Instability | Type | length | tAI | uAUG |  |  |  |  | 0.607263 |
| CAI | Start | Stop | Type | length | tAI |  |  |  |  | 0.617541 |
| CAI | Start | Stop | Type | length | uAUG |  |  |  |  | 0.617998 |
| CAI | Start | Stop | Type | tAI | uAUG |  |  |  |  | 0.61187 |
| CAI | Start | Stop | length | tAI | uAUG |  |  |  |  | 0.612638 |
| CAI | Start | Type | length | tAI | uAUG |  |  |  |  | 0.613807 |
| CAI | Stop | Type | length | tAI | uAUG |  |  |  |  | 0.617692 |
| Half-life | Instability | Start | Stop | Type | length |  |  |  |  | 0.622749 |
| Half-life | Instability | Start | Stop | Type | tAI |  |  |  |  | 0.61807 |
| Half-life | Instability | Start | Stop | Type | uAUG |  |  |  |  | 0.617545 |
| Half-life | Instability | Start | Stop | length | tAI |  |  |  |  | 0.615192 |
| Half-life | Instability | Start | Stop | length | uAUG |  |  |  |  | 0.618541 |
| Half-life | Instability | Start | Stop | tAI | uAUG |  |  |  |  | 0.61185 |
| Half-life | Instability | Start | Type | length | tAI |  |  |  |  | 0.615836 |
| Half-life | Instability | Start | Type | length | uAUG |  |  |  |  | 0.614983 |
| Half-life | Instability | Start | Type | tAI | uAUG |  |  |  |  | 0.613509 |
| Half-life | Instability | Start | length | tAI | uAUG |  |  |  |  | 0.61001 |
| Half-life | Instability | Stop | Type | length | tAI |  |  |  |  | 0.618358 |
| Half-life | Instability | Stop | Type | length | uAUG |  |  |  |  | 0.618791 |
| Half-life | Instability | Stop | Type | tAI | uAUG |  |  |  |  | 0.612903 |
| Half-life | Instability | Stop | length | tAI | uAUG |  |  |  |  | 0.612881 |
| Half-life | Instability | Type | length | tAI | uAUG |  |  |  |  | 0.61308 |
| Half-life | Start | Stop | Type | length | tAI |  |  |  |  | 0.622323 |
| Half-life | Start | Stop | Type | length | uAUG |  |  |  |  | 0.624909 |
| Half-life | Start | Stop | Type | tAI | uAUG |  |  |  |  | 0.618671 |
| Half-life | Start | Stop | length | tAI | uAUG |  |  |  |  | 0.617214 |
| Half-life | Start | Type | length | tAI | uAUG |  |  |  |  | 0.619162 |
| Half-life | Stop | Type | length | tAI | uAUG |  |  |  |  | 0.620036 |
| Instability | Start | Stop | Type | length | tAI |  |  |  |  | 0.61962 |
| Instability | Start | Stop | Type | length | uAUG |  |  |  |  | 0.622723 |
| Instability | Start | Stop | Type | tAI | uAUG |  |  |  |  | 0.615733 |
| Instability | Start | Stop | length | tAI | uAUG |  |  |  |  | 0.615102 |
| Instability | Start | Type | length | tAI | uAUG |  |  |  |  | 0.616722 |
| Instability | Stop | Type | length | tAI | uAUG |  |  |  |  | 0.617924 |
| Start | Stop | Type | length | tAI | uAUG |  |  |  |  | 0.62242 |
| +1 to +30 | CAI | Half-life | Instability | Start | Stop | Type |  |  |  | 0.608431 |
| +1 to +30 | CAI | Half-life | Instability | Start | Stop | length |  |  |  | 0.611075 |
| +1 to +30 | CAI | Half-life | Instability | Start | Stop | tAI |  |  |  | 0.604533 |
| +1 to +30 | CAI | Half-life | Instability | Start | Stop | uAUG |  |  |  | 0.601428 |
| +1 to +30 | CAI | Half-life | Instability | Start | Type | length |  |  |  | 0.610823 |
| +1 to +30 | CAI | Half-life | Instability | Start | Type | tAI |  |  |  | 0.605167 |
| +1 to +30 | CAI | Half-life | Instability | Start | Type | uAUG |  |  |  | 0.604212 |
| +1 to +30 | CAI | Half-life | Instability | Start | length | tAI |  |  |  | 0.605873 |
| +1 to +30 | CAI | Half-life | Instability | Start | length | uAUG |  |  |  | 0.606727 |
| +1 to +30 | CAI | Half-life | Instability | Start | tAI | uAUG |  |  |  | 0.600255 |
| +1 to +30 | CAI | Half-life | Instability | Stop | Type | length |  |  |  | 0.612661 |
| +1 to +30 | CAI | Half-life | Instability | Stop | Type | tAI |  |  |  | 0.606404 |
| +1 to +30 | CAI | Half-life | Instability | Stop | Type | uAUG |  |  |  | 0.602798 |
| +1 to +30 | CAI | Half-life | Instability | Stop | length | tAI |  |  |  | 0.604355 |
| +1 to +30 | CAI | Half-life | Instability | Stop | length | uAUG |  |  |  | 0.606562 |
| +1 to +30 | CAI | Half-life | Instability | Stop | tAI | uAUG |  |  |  | 0.601128 |
| +1 to +30 | CAI | Half-life | Instability | Type | length | tAI |  |  |  | 0.605467 |
| +1 to +30 | CAI | Half-life | Instability | Type | length | uAUG |  |  |  | 0.60515 |
| +1 to +30 | CAI | Half-life | Instability | Type | tAI | uAUG |  |  |  | 0.602259 |
| +1 to +30 | CAI | Half-life | Instability | length | tAI | uAUG |  |  |  | 0.600494 |
| +1 to +30 | CAI | Half-life | Start | Stop | Type | length |  |  |  | 0.618932 |
| +1 to +30 | CAI | Half-life | Start | Stop | Type | tAI |  |  |  | 0.609601 |
| +1 to +30 | CAI | Half-life | Start | Stop | Type | uAUG |  |  |  | 0.611381 |
| +1 to +30 | CAI | Half-life | Start | Stop | length | tAI |  |  |  | 0.610566 |
| +1 to +30 | CAI | Half-life | Start | Stop | length | uAUG |  |  |  | 0.610944 |
| +1 to +30 | CAI | Half-life | Start | Stop | tAI | uAUG |  |  |  | 0.602702 |
| +1 to +30 | CAI | Half-life | Start | Type | length | tAI |  |  |  | 0.613832 |
| +1 to +30 | CAI | Half-life | Start | Type | length | uAUG |  |  |  | 0.615095 |
| +1 to +30 | CAI | Half-life | Start | Type | tAI | uAUG |  |  |  | 0.604757 |
| +1 to +30 | CAI | Half-life | Start | length | tAI | uAUG |  |  |  | 0.607372 |
| +1 to +30 | CAI | Half-life | Stop | Type | length | tAI |  |  |  | 0.614557 |
| +1 to +30 | CAI | Half-life | Stop | Type | length | uAUG |  |  |  | 0.614208 |
| +1 to +30 | CAI | Half-life | Stop | Type | tAI | uAUG |  |  |  | 0.609246 |
| +1 to +30 | CAI | Half-life | Stop | length | tAI | uAUG |  |  |  | 0.607638 |
| +1 to +30 | CAI | Half-life | Type | length | tAI | uAUG |  |  |  | 0.607233 |
| +1 to +30 | CAI | Instability | Start | Stop | Type | length |  |  |  | 0.617123 |
| +1 to +30 | CAI | Instability | Start | Stop | Type | tAI |  |  |  | 0.608463 |
| +1 to +30 | CAI | Instability | Start | Stop | Type | uAUG |  |  |  | 0.607042 |
| +1 to +30 | CAI | Instability | Start | Stop | length | tAI |  |  |  | 0.611068 |
| +1 to +30 | CAI | Instability | Start | Stop | length | uAUG |  |  |  | 0.612246 |
| +1 to +30 | CAI | Instability | Start | Stop | tAI | uAUG |  |  |  | 0.604269 |
| +1 to +30 | CAI | Instability | Start | Type | length | tAI |  |  |  | 0.612084 |
| +1 to +30 | CAI | Instability | Start | Type | length | uAUG |  |  |  | 0.612363 |
| +1 to +30 | CAI | Instability | Start | Type | tAI | uAUG |  |  |  | 0.606237 |
| +1 to +30 | CAI | Instability | Start | length | tAI | uAUG |  |  |  | 0.606691 |
| +1 to +30 | CAI | Instability | Stop | Type | length | tAI |  |  |  | 0.613556 |
| +1 to +30 | CAI | Instability | Stop | Type | length | uAUG |  |  |  | 0.610583 |
| +1 to +30 | CAI | Instability | Stop | Type | tAI | uAUG |  |  |  | 0.605004 |
| +1 to +30 | CAI | Instability | Stop | length | tAI | uAUG |  |  |  | 0.607618 |
| +1 to +30 | CAI | Instability | Type | length | tAI | uAUG |  |  |  | 0.607829 |
| +1 to +30 | CAI | Start | Stop | Type | length | tAI |  |  |  | 0.617524 |
| +1 to +30 | CAI | Start | Stop | Type | length | uAUG |  |  |  | 0.617466 |
| +1 to +30 | CAI | Start | Stop | Type | tAI | uAUG |  |  |  | 0.61216 |
| +1 to +30 | CAI | Start | Stop | length | tAI | uAUG |  |  |  | 0.613108 |
| +1 to +30 | CAI | Start | Type | length | tAI | uAUG |  |  |  | 0.614713 |
| +1 to +30 | CAI | Stop | Type | length | tAI | uAUG |  |  |  | 0.617699 |
| +1 to +30 | Half-life | Instability | Start | Stop | Type | length |  |  |  | 0.622935 |
| +1 to +30 | Half-life | Instability | Start | Stop | Type | tAI |  |  |  | 0.617999 |
| +1 to +30 | Half-life | Instability | Start | Stop | Type | uAUG |  |  |  | 0.617662 |
| +1 to +30 | Half-life | Instability | Start | Stop | length | tAI |  |  |  | 0.615929 |
| +1 to +30 | Half-life | Instability | Start | Stop | length | uAUG |  |  |  | 0.618944 |
| +1 to +30 | Half-life | Instability | Start | Stop | tAI | uAUG |  |  |  | 0.612133 |
| +1 to +30 | Half-life | Instability | Start | Type | length | tAI |  |  |  | 0.61612 |
| +1 to +30 | Half-life | Instability | Start | Type | length | uAUG |  |  |  | 0.614885 |
| +1 to +30 | Half-life | Instability | Start | Type | tAI | uAUG |  |  |  | 0.61285 |
| +1 to +30 | Half-life | Instability | Start | length | tAI | uAUG |  |  |  | 0.610615 |
| +1 to +30 | Half-life | Instability | Stop | Type | length | tAI |  |  |  | 0.618414 |
| +1 to +30 | Half-life | Instability | Stop | Type | length | uAUG |  |  |  | 0.61861 |
| +1 to +30 | Half-life | Instability | Stop | Type | tAI | uAUG |  |  |  | 0.6129 |
| +1 to +30 | Half-life | Instability | Stop | length | tAI | uAUG |  |  |  | 0.612919 |
| +1 to +30 | Half-life | Instability | Type | length | tAI | uAUG |  |  |  | 0.612223 |
| +1 to +30 | Half-life | Start | Stop | Type | length | tAI |  |  |  | 0.622565 |
| +1 to +30 | Half-life | Start | Stop | Type | length | uAUG |  |  |  | 0.624157 |
| +1 to +30 | Half-life | Start | Stop | Type | tAI | uAUG |  |  |  | 0.619097 |
| +1 to +30 | Half-life | Start | Stop | length | tAI | uAUG |  |  |  | 0.617137 |
| +1 to +30 | Half-life | Start | Type | length | tAI | uAUG |  |  |  | 0.618829 |
| +1 to +30 | Half-life | Stop | Type | length | tAI | uAUG |  |  |  | 0.619896 |
| +1 to +30 | Instability | Start | Stop | Type | length | tAI |  |  |  | 0.619536 |
| +1 to +30 | Instability | Start | Stop | Type | length | uAUG |  |  |  | 0.622874 |
| +1 to +30 | Instability | Start | Stop | Type | tAI | uAUG |  |  |  | 0.615833 |
| +1 to +30 | Instability | Start | Stop | length | tAI | uAUG |  |  |  | 0.615882 |
| +1 to +30 | Instability | Start | Type | length | tAI | uAUG |  |  |  | 0.616791 |
| +1 to +30 | Instability | Stop | Type | length | tAI | uAUG |  |  |  | 0.617583 |
| +1 to +30 | Start | Stop | Type | length | tAI | uAUG |  |  |  | 0.621961 |
| CAI | Half-life | Instability | Start | Stop | Type | length |  |  |  | 0.615772 |
| CAI | Half-life | Instability | Start | Stop | Type | tAI |  |  |  | 0.606202 |
| CAI | Half-life | Instability | Start | Stop | Type | uAUG |  |  |  | 0.605396 |
| CAI | Half-life | Instability | Start | Stop | length | tAI |  |  |  | 0.606697 |
| CAI | Half-life | Instability | Start | Stop | length | uAUG |  |  |  | 0.60929 |
| CAI | Half-life | Instability | Start | Stop | tAI | uAUG |  |  |  | 0.599958 |
| CAI | Half-life | Instability | Start | Type | length | tAI |  |  |  | 0.608982 |
| CAI | Half-life | Instability | Start | Type | length | uAUG |  |  |  | 0.607339 |
| CAI | Half-life | Instability | Start | Type | tAI | uAUG |  |  |  | 0.604368 |
| CAI | Half-life | Instability | Start | length | tAI | uAUG |  |  |  | 0.602423 |
| CAI | Half-life | Instability | Stop | Type | length | tAI |  |  |  | 0.610818 |
| CAI | Half-life | Instability | Stop | Type | length | uAUG |  |  |  | 0.606918 |
| CAI | Half-life | Instability | Stop | Type | tAI | uAUG |  |  |  | 0.601572 |
| CAI | Half-life | Instability | Stop | length | tAI | uAUG |  |  |  | 0.602358 |
| CAI | Half-life | Instability | Type | length | tAI | uAUG |  |  |  | 0.604641 |
| CAI | Half-life | Start | Stop | Type | length | tAI |  |  |  | 0.616364 |
| CAI | Half-life | Start | Stop | Type | length | uAUG |  |  |  | 0.615562 |
| CAI | Half-life | Start | Stop | Type | tAI | uAUG |  |  |  | 0.608262 |
| CAI | Half-life | Start | Stop | length | tAI | uAUG |  |  |  | 0.608836 |
| CAI | Half-life | Start | Type | length | tAI | uAUG |  |  |  | 0.610675 |
| CAI | Half-life | Stop | Type | length | tAI | uAUG |  |  |  | 0.613295 |
| CAI | Instability | Start | Stop | Type | length | tAI |  |  |  | 0.612868 |
| CAI | Instability | Start | Stop | Type | length | uAUG |  |  |  | 0.614687 |
| CAI | Instability | Start | Stop | Type | tAI | uAUG |  |  |  | 0.60647 |
| CAI | Instability | Start | Stop | length | tAI | uAUG |  |  |  | 0.609331 |
| CAI | Instability | Start | Type | length | tAI | uAUG |  |  |  | 0.609059 |
| CAI | Instability | Stop | Type | length | tAI | uAUG |  |  |  | 0.610736 |
| CAI | Start | Stop | Type | length | tAI | uAUG |  |  |  | 0.615355 |
| Half-life | Instability | Start | Stop | Type | length | tAI |  |  |  | 0.618892 |
| Half-life | Instability | Start | Stop | Type | length | uAUG |  |  |  | 0.62269 |
| Half-life | Instability | Start | Stop | Type | tAI | uAUG |  |  |  | 0.614789 |
| Half-life | Instability | Start | Stop | length | tAI | uAUG |  |  |  | 0.614206 |
| Half-life | Instability | Start | Type | length | tAI | uAUG |  |  |  | 0.614678 |
| Half-life | Instability | Stop | Type | length | tAI | uAUG |  |  |  | 0.614746 |
| Half-life | Start | Stop | Type | length | tAI | uAUG |  |  |  | 0.620876 |
| Instability | Start | Stop | Type | length | tAI | uAUG |  |  |  | 0.618534 |
| +1 to +30 | CAI | Half-life | Instability | Start | Stop | Type | length |  |  | 0.615581 |
| +1 to +30 | CAI | Half-life | Instability | Start | Stop | Type | tAI |  |  | 0.606247 |
| +1 to +30 | CAI | Half-life | Instability | Start | Stop | Type | uAUG |  |  | 0.605471 |
| +1 to +30 | CAI | Half-life | Instability | Start | Stop | length | tAI |  |  | 0.607216 |
| +1 to +30 | CAI | Half-life | Instability | Start | Stop | length | uAUG |  |  | 0.610785 |
| +1 to +30 | CAI | Half-life | Instability | Start | Stop | tAI | uAUG |  |  | 0.599514 |
| +1 to +30 | CAI | Half-life | Instability | Start | Type | length | tAI |  |  | 0.608802 |
| +1 to +30 | CAI | Half-life | Instability | Start | Type | length | uAUG |  |  | 0.607458 |
| +1 to +30 | CAI | Half-life | Instability | Start | Type | tAI | uAUG |  |  | 0.603474 |
| +1 to +30 | CAI | Half-life | Instability | Start | length | tAI | uAUG |  |  | 0.603874 |
| +1 to +30 | CAI | Half-life | Instability | Stop | Type | length | tAI |  |  | 0.611455 |
| +1 to +30 | CAI | Half-life | Instability | Stop | Type | length | uAUG |  |  | 0.606744 |
| +1 to +30 | CAI | Half-life | Instability | Stop | Type | tAI | uAUG |  |  | 0.601623 |
| +1 to +30 | CAI | Half-life | Instability | Stop | length | tAI | uAUG |  |  | 0.601781 |
| +1 to +30 | CAI | Half-life | Instability | Type | length | tAI | uAUG |  |  | 0.60343 |
| +1 to +30 | CAI | Half-life | Start | Stop | Type | length | tAI |  |  | 0.616422 |
| +1 to +30 | CAI | Half-life | Start | Stop | Type | length | uAUG |  |  | 0.614585 |
| +1 to +30 | CAI | Half-life | Start | Stop | Type | tAI | uAUG |  |  | 0.607942 |
| +1 to +30 | CAI | Half-life | Start | Stop | length | tAI | uAUG |  |  | 0.609667 |
| +1 to +30 | CAI | Half-life | Start | Type | length | tAI | uAUG |  |  | 0.610236 |
| +1 to +30 | CAI | Half-life | Stop | Type | length | tAI | uAUG |  |  | 0.613308 |
| +1 to +30 | CAI | Instability | Start | Stop | Type | length | tAI |  |  | 0.612827 |
| +1 to +30 | CAI | Instability | Start | Stop | Type | length | uAUG |  |  | 0.615194 |
| +1 to +30 | CAI | Instability | Start | Stop | Type | tAI | uAUG |  |  | 0.606473 |
| +1 to +30 | CAI | Instability | Start | Stop | length | tAI | uAUG |  |  | 0.610634 |
| +1 to +30 | CAI | Instability | Start | Type | length | tAI | uAUG |  |  | 0.609322 |
| +1 to +30 | CAI | Instability | Stop | Type | length | tAI | uAUG |  |  | 0.611508 |
| +1 to +30 | CAI | Start | Stop | Type | length | tAI | uAUG |  |  | 0.615598 |
| +1 to +30 | Half-life | Instability | Start | Stop | Type | length | tAI |  |  | 0.617974 |
| +1 to +30 | Half-life | Instability | Start | Stop | Type | length | uAUG |  |  | 0.622518 |
| +1 to +30 | Half-life | Instability | Start | Stop | Type | tAI | uAUG |  |  | 0.613553 |
| +1 to +30 | Half-life | Instability | Start | Stop | length | tAI | uAUG |  |  | 0.615449 |
| +1 to +30 | Half-life | Instability | Start | Type | length | tAI | uAUG |  |  | 0.61416 |
| +1 to +30 | Half-life | Instability | Stop | Type | length | tAI | uAUG |  |  | 0.614998 |
| +1 to +30 | Half-life | Start | Stop | Type | length | tAI | uAUG |  |  | 0.62058 |
| +1 to +30 | Instability | Start | Stop | Type | length | tAI | uAUG |  |  | 0.61794 |
| CAI | Half-life | Instability | Start | Stop | Type | length | tAI |  |  | 0.612298 |
| CAI | Half-life | Instability | Start | Stop | Type | length | uAUG |  |  | 0.611795 |
| CAI | Half-life | Instability | Start | Stop | Type | tAI | uAUG |  |  | 0.601888 |
| CAI | Half-life | Instability | Start | Stop | length | tAI | uAUG |  |  | 0.604722 |
| CAI | Half-life | Instability | Start | Type | length | tAI | uAUG |  |  | 0.604716 |
| CAI | Half-life | Instability | Stop | Type | length | tAI | uAUG |  |  | 0.606947 |
| CAI | Half-life | Start | Stop | Type | length | tAI | uAUG |  |  | 0.613414 |
| CAI | Instability | Start | Stop | Type | length | tAI | uAUG |  |  | 0.611884 |
| Half-life | Instability | Start | Stop | Type | length | tAI | uAUG |  |  | 0.616553 |
| +1 to +30 | CAI | Half-life | Instability | Start | Stop | Type | length | tAI |  | 0.612753 |
| +1 to +30 | CAI | Half-life | Instability | Start | Stop | Type | length | uAUG |  | 0.611767 |
| +1 to +30 | CAI | Half-life | Instability | Start | Stop | Type | tAI | uAUG |  | 0.602061 |
| +1 to +30 | CAI | Half-life | Instability | Start | Stop | length | tAI | uAUG |  | 0.605462 |
| +1 to +30 | CAI | Half-life | Instability | Start | Type | length | tAI | uAUG |  | 0.604349 |
| +1 to +30 | CAI | Half-life | Instability | Stop | Type | length | tAI | uAUG |  | 0.607798 |
| +1 to +30 | CAI | Half-life | Start | Stop | Type | length | tAI | uAUG |  | 0.613706 |
| +1 to +30 | CAI | Instability | Start | Stop | Type | length | tAI | uAUG |  | 0.612097 |
| +1 to +30 | Half-life | Instability | Start | Stop | Type | length | tAI | uAUG |  | 0.616346 |
| CAI | Half-life | Instability | Start | Stop | Type | length | tAI | uAUG |  | 0.609802 |
| +1 to +30 | CAI | Half-life | Instability | Start | Stop | Type | length | tAI | uAUG | 0.609977 |
